# Identification of a risk model for prognostic and therapeutic prediction in renal cell carcinoma based on infiltrating M0 cells

**DOI:** 10.1038/s41598-024-64207-0

**Published:** 2024-06-11

**Authors:** Shiyong Xin, Junjie Su, Ruixin Li, Qiong Cao, Haojie Wang, Zhihao Wei, Chengliang Wang, Chengdong Zhang

**Affiliations:** 1 Department of Urology, The First Affiliated Hospital and College of Clinical Medicine of Henan University of Science and Technology, No. 636, Guan-lin Rd, Luo-long District, Luoyang, China; 2 Department of Pathology, The Third Affiliated Hospital of Henan University of Science and Technology, Luoyang, 471003 China; 3 Department of Central Laboratory, Zhengzhou University, Luoyang Central Hospital, Luoyang, 471003 China; 4 Department of Pathology, The Yiluo Hospital of Luoyang, The Teaching Hospital of Henan University of Science and Technology, Luoyang, 471023 China; 5 Department of Urology, Shangcheng County People’s Hospital, Xinyang, 464000 China; 6 Department of Urology, Xinxiang First People’s Hospital, Xinxiang, 453000 China

**Keywords:** Renal cell carcinoma, Risk model, Immunotherapy, M0, Prognosis, Cancer, Computational biology and bioinformatics

## Abstract

The tumor microenvironment (TME) comprises immune-infiltrating cells that are closely linked to tumor development. By screening and analyzing genes associated with tumor-infiltrating M0 cells, we developed a risk model to provide therapeutic and prognostic guidance in clear cell renal cell carcinoma (ccRCC). First, the infiltration abundance of each immune cell type and its correlation with patient prognosis were analyzed. After assessing the potential link between the depth of immune cell infiltration and prognosis, we screened the infiltrating M0 cells to establish a risk model centered on three key genes (TMEN174, LRRC19, and SAA1). The correlation analysis indicated a positive correlation between the risk score and various stages of the tumor immune cycle, including B-cell recruitment. Furthermore, the risk score was positively correlated with CD8 expression and several popular immune checkpoints (ICs) (TIGIT, CTLA4, CD274, LAG3, and PDCD1). Additionally, the high-risk group (HRG) had higher scores for tumor immune dysfunction and exclusion (TIDE) and exclusion than the low-risk group (LRG). Importantly, the risk score was negatively correlated with the immunotherapy-related pathway enrichment scores, and the LRG showed a greater therapeutic benefit than the HRG. Differences in sensitivity to targeted drugs between the HRG and LRG were analyzed. For commonly used targeted drugs in RCC, including axitinib, pazopanib, temsirolimus, and sunitinib, LRG had lower IC50 values, indicating increased sensitivity. Finally, immunohistochemistry results of 66 paraffin-embedded specimens indicated that SAA1 was strongly expressed in the tumor samples and was associated with tumor metastasis, stage, and grade. SAA1 was found to have a significant pro-tumorigenic effect by experimental validation. In summary, these data confirmed that tumor-infiltrating M0 cells play a key role in the prognosis and treatment of patients with ccRCC. This discovery offers new insights and directions for the prognostic prediction and treatment of ccRCC.

## Introduction

Renal cell carcinoma (RCC) is a prevalent malignant tumor of the urinary system, representing 2–3% of all malignancies in adults^1^. In 2018, more than 350,000 individuals worldwide were diagnosed with RCC, resulting in over 170,000 deaths^2,3^. Clear cell renal cell carcinoma (ccRCC), comprising 70–80% of all kidney cancers, is the most prevalent subtype of RCC^4^. Patients with ccRCC have higher tumor recurrence and metastasis rates than those with other renal cancer subtypes and generally exhibit poor prognoses^5,6^. Due to the high metastatic potential of renal cancer cells, patients are typically diagnosed at intermediate or advanced stages^7^. For newly diagnosed early RCC, the primary modality is surgical treatment, ranging from open surgery to laparoscopic minimally invasive surgery and robotic surgery, which brings significant advantages to patients. However, the prognosis of advanced metastatic RCC is poor and often lacks surgical indications; therefore, systematic therapy has become the main treatment option. Currently, targeted drugs used for the treatment of advanced RCC mainly belong to two categories: tyrosine kinase inhibitors (TKI), such as sunitinib, pazopanib, and acitinib^8^. The other group includes mammalian target of rapamycin (mTOR) inhibitors, such as everolimus and tisirolimus^9^. The advent of immunotherapy (alone or in combination) has revolutionized the treatment of advanced RCC. Immune checkpoint inhibitors (ICI) are effective therapeutic agents for various hematological and solid tumors^10^, including programmed cell death 1 (PD-1), programmed cell death ligand 1 (PD-L1), and cytotoxic T lymphocyte-associated antigen-4 (CTLA-4)^10–12^. However, prognostic and predictive biomarkers for immunotherapy in clinical practice are lacking. Additionally, ICI monotherapy and combination therapy were shown to be associated with higher levels of transaminemia in patients^11^. Patients who exhibit a poor response or resistance to immunotherapy face a risk of death, underscoring the need for effective solutions. Therefore, there is an urgent need to identify effective biomarkers to tailor treatment based on the patient and tumor characteristics, avoid unnecessary toxicity, and improve the patient’s quality of life^13^.

Previous research has demonstrated that tumor progression is not only related to the growth of the tumor itself but also to the tumor microenvironment (TME)^14,15^, which is a complex, highly heterogeneous, and dynamic system^16,17^. Immune cells infiltrating the TME contribute greatly to tumor development and assist tumor cells in their immune escape abilities^18^. Tumor cells can also interfere with the immune system by inducing and/or attracting immunosuppressive cells (mechanisms that prevent excessive inflammation or autoimmune disease) that impede antitumor activity of the immune system. Tumor-associated macrophages (TAM), Tregs, Myeloid-derived Suppressor Cells (MDSCs), Mast Cells, Neutrophils, B lymphocytes, and other immunosuppressive cells secrete several signaling molecules, including growth factors, chemokines, and cytokines that promote tumor progression. Secretion of angiogenic and/or invasive stroma-degrading enzymes, including MMP9 and other MMP molecules, induces and sustains tumor growth^19^. Attracted by chemokine ligands secreted by tumors and macrophages, Tregs infiltrate tumors and inhibit a variety of immunoactive cells, including CD8+ T cells, NK cells, B cells, and antigen-presenting cells^20^. In the TME, Tregs are activated by tumor-specific antigens or autoantigens released by tumor death, selectively inhibiting the activation of tumor-associated antigen (TAA)-specific effector T cells through a variety of mechanisms. The primary mechanism of tumor immune escape appears to be immunosuppression in the TME mediated by CD4, CD25–FoxP3–Tregs or other types of suppressor cells^21^. Phenotypic transformation of NK cells can inhibit immune cell function and promote tumor proliferation by expressing high levels of vascular endothelial growth factor (VEGF)^22^. High levels of TGF-β and anoxia in the TME may also induce NK cell transformation. CD8+ T cells play a major role in immunotherapy, mainly by interfering with their activity in treating patients with tumors^23^. In many tumors, the number of CD8+ T cells is reduced or inhibited by different mechanisms to induce immune escape. For example, TIRIB3 can reduce the infiltration of CD8+ T cells in colorectal cancer^23^, inhibit the response of CD8+ T cells to N6-methyladenosine-modified circIGF2BP3 in non-small cell lung carcinoma^24^, and promote CD8+ T cell rejection in Progranulin in breast cancer. These factors all induce immune escape^25^. TAM and dendritic cells (DC) in the TME can also inhibit tumor immunity^26,27^. M1-type macrophages promote antitumor immunity by presenting antigens, producing pro-inflammatory factors, and phagocytizing tumor cells, whereas M2-type macrophages promote tumor progression by upregulating cytokine secretion and protein expression. M0 macrophages have traditionally been thought to be precursors of polarized macrophages and have no specific functions. M2 macrophages enhance proliferation, migration, invasion, and epithelial–mesenchymal transition (EMT) of ccRCC cell line^28^. However, it has been found that macrophages infiltrating glioma tissue maintain a continuum between the M1 and M2 phenotypes, similar to M0 macrophages^29^. Moreover, the authors found that M0 macrophages in gliomas are associated with high-level tumors and poor prognosis, suggesting that M0 macrophages may play a tumorigenic role in tumor development^30^. Few studies have focused on the role of M0 macrophages in ccRCC tumorigenesis and development. ccRCC is sensitive to radiotherapy and chemotherapy and can benefit from immunotherapy. Therefore, this study analyzed the relationship between infiltrating M0 cells in the TME of ccRCC, patient prognosis, and immunotherapy.

This study focused on comprehensively characterizing immune cell infiltration in ccRCC and identifying immune cells and models relevant for prognosis and treatment. We investigated the abundance of immune-associated cells in ccRCC and evaluated their association with prognosis. We analyzed and filtered the genes in immune cells that had the most favorable prognosis between the groups with high and low infiltration. Additionally, risk models associated with immune cells were developed using key differentially expressed genes (DEGs). The sample was then divided into the high-risk group (HRG) and low-risk group (LRG) based on the risk score, and the survival status of the two groups was observed separately. To investigate the relationship between the risk score and immune characteristics, expression of ICs, IC50 values of targeted drugs, and immune efficacy, we performed a correlation analysis. Finally, immunohistochemistry was performed to verify the expression and functional roles of SAA1. This study investigated the potential role of M0 cells in the TME of ccRCC and the value of risk models for guiding patient medication and prognosis.

## Methods

All methods were carried out in accordance with relevant guidelines and regulations.

### Access to data

ccRCC sample data were obtained from The Cancer Genome Atlas (TCGA) (https://portal.gdc.cancer.gov/analysis_page?app=Downloads), Gene Expression Omnibus (GEO) database (cohort number: GSE40435) (https://www.ncbi.nlm.nih.gov/geo/query/acc.cgi?acc=GSE40435), and ArrayExpress database (E-MTAB-1980) (https://www.ebi.ac.uk/biostudies/arrayexpress/studies?query=E-MTAB-1980). This comprehensive dataset encompasses RNA sequencing data, along with information on age, sex, and pathological stage, sourced from 541 ccRCC samples from TCGA, additional 101 samples from GEO, and 98 samples from ArrayExpress. Paraffin-embedded specimens of 66 patients with RCC (41 males and 25 females) and 18 normal renal tissue specimens and their corresponding clinical information were collected from the First Affiliated Hospital of Henan University of Science and Technology from January 1, 2020, to December 31, 2022. The 2009 TNM staging system was used for tumor staging. The tumor grade was determined according to the Fuhrman grading system (well-differentiated, grades 1 and 2; moderately differentiated, grade 3; and poorly differentiated, grade 4).

### Analysis of immune cells

Firstly, we collected marker genes of 22 immune cells from the literature^31^. The abundance of immune cell infiltration in each sample was calculated according to the gene expression matrix (FPKM) through the R package CIBERSORT, setting perm = 1000. According to the infiltration abundance of each type of immune cells, the samples were divided into high and low infiltration groups, and then the survival differences between the two groups were compared by Kaplan–Meier (KM) survival analysis (Log-rank). p < 0.05 and |HR-1| > 0.5 were used to screen immune cells highly related to ccRCC.

### DEGs analysis

The ccRCC cohort was categorized into high- and low-infiltration groups based on the extent of immune cell infiltration. The DEGs were analyzed using the Limma software package (version 4.22). We set the threshold of significance at p < 0.05 and |log fold change (FC)| > = 1.

### Functional analysis

The DEGs identified in the previous step were entered into the STRING database, and a confidence threshold > 0.4 was set to construct the network. Subsequently, non-interacting genes were eliminated, fine-tuned, and embellished using Cytoscape (version 3.9.1). To further investigate the biological functions of these DEGs, we used the DAVID database for the enrichment analysis of Kyoto Encyclopedia of Genes and Genomes (KEGG)^32–34^ pathways, biological processes (BP), cellular components (CC), and molecular functions (MF). We considered terms with a p-value < 0.05 to be significant.

### Development and validation of the immune-risk score

Univariate Cox regression was conducted to screen for prognostically relevant genes in the training cohort of ccRCC patients using the R survival package. To resolve issues related to collinearity and overfitting, the “glmnet” package (version 4.22) was employed to perform LASSO analysis on survival-related genes (p < 0.05) to filter predictors. Immunity-related risk models (IRRM) were constructed using multivariate Cox regression analysis based on the LASSO analysis results using the R package survival and survminer. Risk scores for all samples were calculated based on the weighted estimates of the expression of each gene. The ccRCC samples in both the training and validation cohorts were segregated into the LRG and HRG, respectively, based on the median risk score. KM survival analysis was used to investigate the prognostic value of survival in both the training and validation cohorts, with p < 0.05 as significance threshold. Additionally, the prognostic ability of the risk score was assessed using receiver operating characteristic (ROC) curves. We set the threshold of significance at p < 0.05 and AUC ≥ 0.65.

### Creating a nomogram

Initially, we conducted Cox regression analyses of the selected genes to screen for independent prognostic factors for ccRCC. Therefore, we developed a nomogram to predict the likelihood of survival in patients with ccRCC. The method visually displays a set of scoring criteria derived from the magnitude of the regression coefficient for each variable and assigns a score to the level of each value for the respective variable. After calculating the total score for each patient using the method described above, we can approximate the prognostic probability for each patient using the function between the total score and prognostic probability^35^. Finally, to assess the efficacy of the nomogram, we generated corresponding calibration curves.

### Calculation of enrichment scores for different genetic traits

A group of gene signatures was used to predict reactions to immunotherapy^36^. Furthermore, by tracking the tumor immunophenotype (TIP) (http://biocc.hrbmu.edu.cn/TIP/index.jsp), we obtained some gene features associated with the cancer immune cycle^37^. Pearson correlations were used to analyze the association between risk scores and gene features associated with cancer immune cycles using the R packages ggplot2 and ggcor.

### Analysis of tumor immune dysfunction and exclusion (TIDE)

TIDE is primarily used to predict a tumor’s ability to survive an immune system attack based on its gene expression profile^38^. To calculate relevant immune parameters, such as microsatellite instability (MSI), TIDE, and CD8 and CD274 scores, and to predict ICIs response rates, we uploaded the gene expression files of the ccRCC samples to the TIDE website (http://tide.dfci). Unpaired *t*-tests were performed for tumor exclusion and TIDE scores between the HRG and LRG, while the association between CD8, CD274, and the risk score was analyzed using Spearman's correlation. A statistical significance level of p < 0.05 was set for all analyses.

### Correlation between risk model and immunotherapy biomarkers

This study aimed to investigate the association between the immune risk score and IC. In addition, Pearson’s correlation was used to analyze the relationship between the risk score and five critical ICs (TIGIT, PDCD1, CTLA4, LAG3, and CD274) using the R packages circlize and corrplot. Additionally, we analyzed the differences in TIGIT, PDCD1, CTLA4, and LAG3 expression between the HRG and LRG.

### Drug sensitivity analyses

The Oncoprecdict R package was used to predict drug responses among cancer patients in vivo^39^. It works by matching the expression data of the sample genes with the IC50 of the tumor cell line to the drug in the Genomics of Drug Sensitivity in Cancer. In this study, 283 drug sensitivities were compared between the HRG and LRG using an unpaired *t*-test set at p < 0.05.

### Cell treatment

The cell lines (HEK-293, 786-O, 769-P, and THP-1) were obtained from HyCyteTM (Hai Xing, China). SAA1 siRNA was designed and synthesized by GenePharma and transfected according to the manufacturer's instructions. THP-1 cells in the logarithmic growth phase were treated with 100 ng/mL PMA and cultured for 48 h. THP-1 cells differentiated into wall-adherent macrophages.

### Immunohistochemistry (IHC)

Paraffin sections were deparaffinized by heating. The sections were hydrated using a gradient of alcohol concentration. Sections were heated and boiled in a pot of antigen recovery solution for 15 min and then treated with 3% hydrogen peroxide and PBS. After drying, the primary antibody (1:400, Bioss bs-19359R) was added and incubated for 12 h. The next day, the primary antibodies were washed three times with PBS. Secondary antibody was added and incubated for 30 min before washing with PBS. Drops of DAB were added and observed. Staining was stopped if necessary, and the sections were rinsed with clean water. Finally, the dye was counterstained with hematoxylin.

### Western blot

Cells were treated with RIPA lysis buffer (Solarbio) and centrifuged at 12,000 rpm for 30 min. The supernatants were collected for western blot analysis. Proteins in the supernatant were separated by SDS-PAGE (12%, Beyotime). The separated proteins were transferred to a membrane and sealed with nonfat dry milk. Primary antibody (abcam ab200485) was added and kept overnight at 4 °C. The following day, the primary antibody was washed off and incubated with a secondary antibody.

### Proliferation experiment

The treated cells were seeded in 6-well plates at a density of 3 × 10^6^ cells/well, and the cell state was stabilized. Experimental treatments were performed using the BeyoClickTM EDU-555 Cell Proliferation Assay Kit (Beyotime).

The prepared cells were transferred to 6-well plates at a density of 1000 cells per well. After 2 weeks of incubation, photographs were taken for observation.

### Transwell

Matrigel gel, diluted 1:8 with serum-free cell culture medium, and 60 μL was taken and added evenly to the upper chamber, which was incubated for 3 h at 37 °C to polymerize the matrix gel into a thin film. Cells (5 × 10^4^) were suspended in 150 μL serum-free medium and added to the upper chamber. 600 μL of medium was added to the lower chamber. After 48 h of incubation, the cells migrating across the membrane were fixed with formaldehyde and stained with 0.1% crystal violet. The migration assay did not require the Matrigel gel and the rest of the procedure was the same as that used for invasion.

### Immunostaining evaluation

Staining results were evaluated by two professional investigators. The positive signal for SAA1 protein was localized in the nucleus and cytoplasm. Based on the proportion of positively stained cells in the total number of cells, positive cells were categorized and classified into four groups: 0 (0%), + 1 (1–5%), + 2 (5–10%), and + 3 (> 10%). SAA1 expression scores were 0 (negative), + 1 (weak), + 2 (moderate), and + 3 (strong). The two scores were multiplied to obtain the final score.

### Statistical analysis

All data were analyzed using the R software (v4.1.3), and a *t*-test was used to analyze normally distributed data. Student’s *t*-test was used to compare two independent samples. Pearson’s correlation was used to analyze the correlation between the risk score, immune checkpoints, and MSI. The relationship between SAA1 expression and the clinicopathological features of ccRCC was analyzed using the Mann–Whitney *U* test. Statistical significance was set at p < 0.05.

### Ethics approval and consent to participate

The ethics involved in this study was approved by the Ethics Committee of the First Affiliated Hospital of Henan University of Science and Technology. Confirmation that informed consent was obtained from all subjects and/or their legal guardian(s).

## Results

### Screening for immune cells associated with ccRCC prognosis

**Figure 1:**
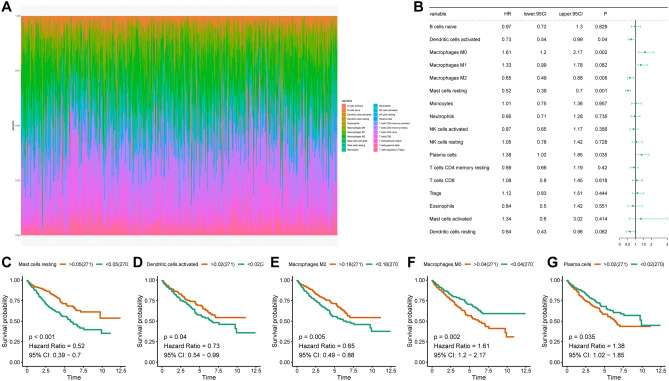
Highly infiltrative M0 cells are associated with poor prognosis in ccRCC. (**A**) Gene expression matrices of ccRCC tissue in TCGA were converted to 22 immune cell infiltration abundances by CIBERSORT. (**B**) The effect of 77 significant immune cells on survival in ccRCC was analysed using the KM survival analysis. (**C**–**G**) Results of KM survival analysis of Mast cells, Dendritic cells, Macrophages M2, Macrophages M0, and Plasma cells in ccRCC.

Initially, the ccRCC gene expression profile was translated into the degree of infiltration of 22 immune cells using CIBERSORT (Fig. 1A). Subsequently, we conducted KM survival analysis by the log-rank test and discovered that the prognosis of ccRCC patients was associated with five immune cells: mast cells (HR = 0.52, 95% [CI] = 0.39–0.7), dendritic cells (HR = 0.73, 95% [CI] = 0.54–0.99), M2 macrophages (HR = 0.65, 95% [CI] = 0.49–0.88), M0 macrophages (HR = 1.61, 95% [CI] = 1.2–2.17), and plasma cells (HR = 1.38, 95% [CI] = 1.02–1.85) (Fig. 1B). We then plotted survival curves to analyze the survival differences between the high- and low-infiltration groups of the five immune cell types. Our results demonstrated that patients with a high infiltration of M0 and plasma cells had a significantly poorer prognosis. In contrast, the other three cell types (resting mast cells, activated dendritic cells activated and macrophages M2) had significantly better prognoses in the high infiltration group than in the low infiltration group (Fig. 1C–G). In contrast, the other cell types did not show any significant differences, except for CD4+ memory-activated T cells, follicular helper T cells, and gamma delta T cells (Fig. S1A–N).

### Analysis of DEGs between highly infiltrated and lowly infiltrated M0 cells

**Figure 2:**
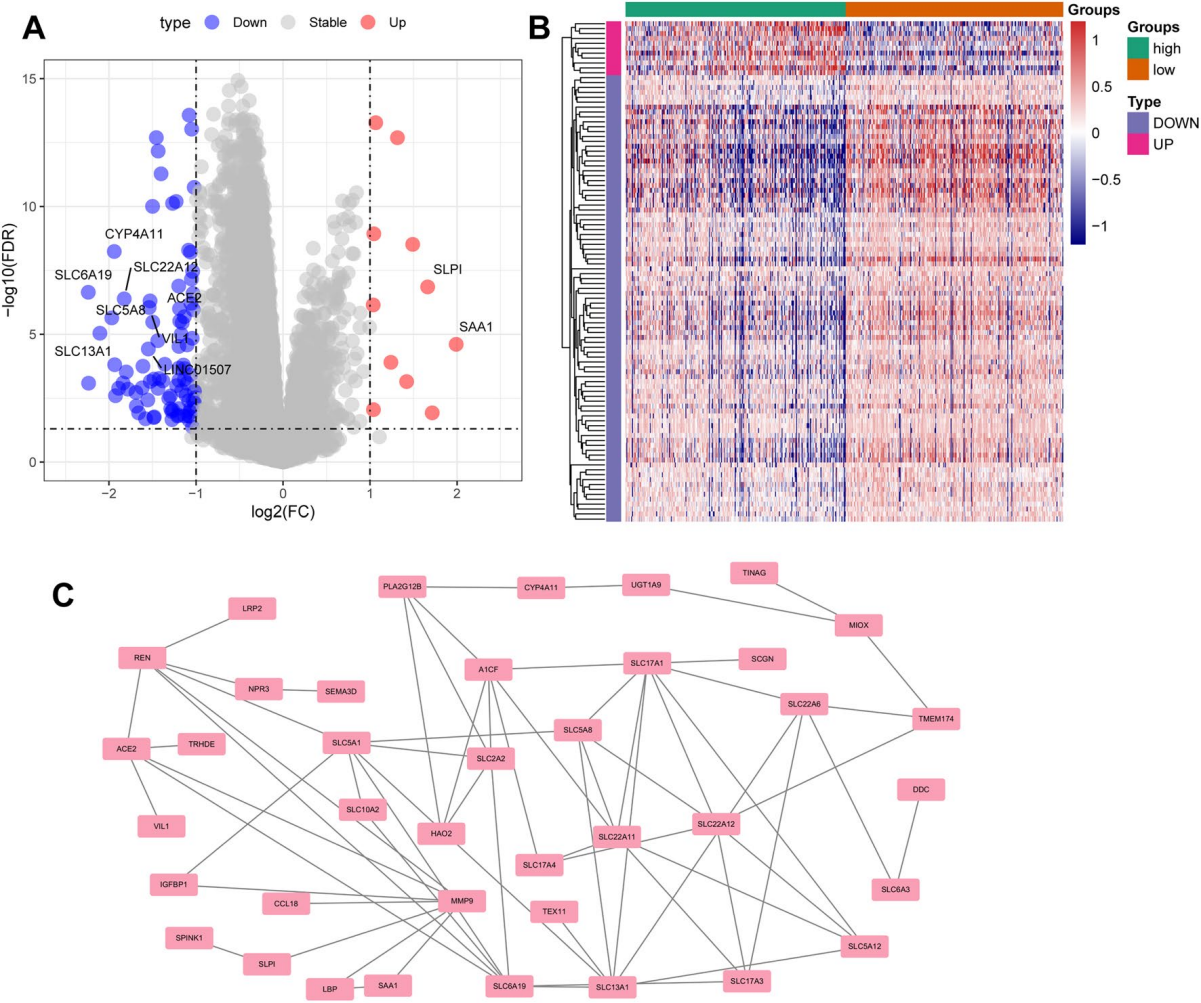
Identification of DEGs between the low and high infiltration groups of M0 cells in ccRCC. (**A**) The volcano plot was used visualize the DEGs (the red colour indicates upregulated genes, while blue colour indicates downregulated genes). (**B**) The heatmap plot was used visualize the DEGs. (**C**) The PPI network revealed associations between 39 genes associated with M0 macrophage infiltration.

Expression data from ccRCC samples in the training cohort were translated to infiltration levels of 22 immune cells using CIBERSORT. Subsequently, based on the median level of M0 cell infiltration, the samples were categorized into high- and low-infiltration groups, resulting in 102 DEGs, including 11 upregulated and 91 downregulated genes (Fig. 2A,B) (Supplementary Dataset File 1). Most DEGs were highly expressed in highly infiltrated M0 cells as shown in Fig. 2B. Furthermore, the DEGs of the other four cell types were analyzed and found to be present only in resting mast cells, albeit in small numbers (Fig. S2A,B). Finally, a PPI network was constructed via PPI analysis, which indicated that 39 DEGs were associated with other genes (Fig. 2C).

### Prediction of DEG function from M0 cells

**Figure 3:**
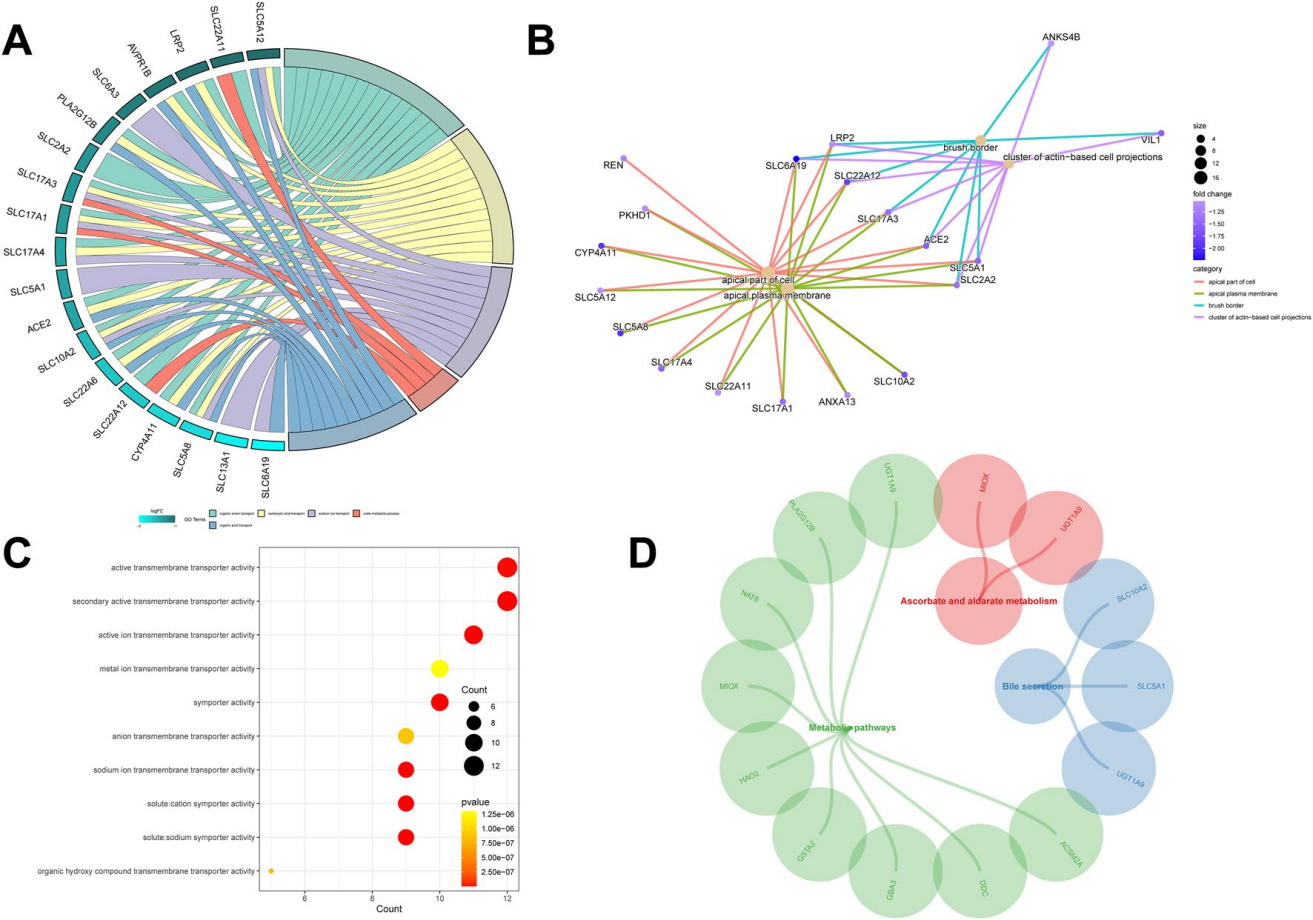
Functional analysis of DEGs in M0 cells. (**A**–**D**) KEGG and GO analysis of 39 genes associated with M0 macrophages.

In total, 102 DEGs related to M0 cells were subjected to GO and KEGG enrichment analyses to identify the pathways in which they were enriched. The findings demonstrated that these DEGs were predominantly clustered in pathways associated with “neutrophil chemotaxis,” “glucose transmembrane transport,” “insulin-like growth factor I binding,” and “metabolic pathways” (Fig. 3A–D). In addition, we present the results of GO analyses (BP, MF, and CC) using chord and line plots (Fig. S3A–C). Our findings suggest that M0-related DEGs are predominantly involved in metabolic pathways.

### Risk model based on M0-related DEGs

**Figure 4:**
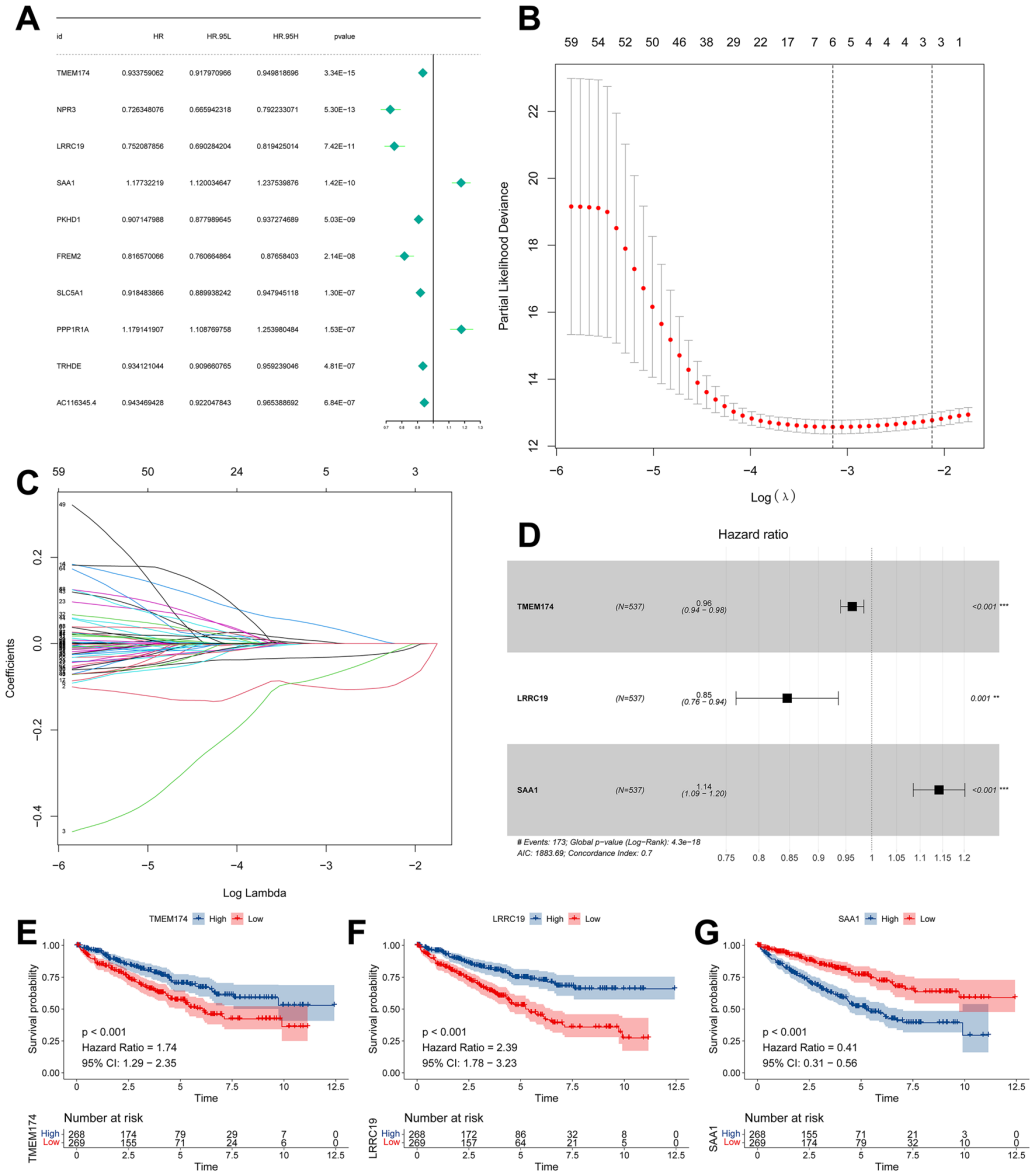
Develop an immunization riskscore. (**A**) Univariate Cox regression analysis was conducted on 102 genes associated with M0 macrophage infiltration. (**B**,**C**) Lasso analysis of other key genes linked to ccRCC survival. (**D**) A multivariate Cox regression analysis was performed on six genes that are linked to M0 macrophage infiltration. (**E**–**G**) Survival analyses showed survival differences between the low and high expression groups of TMEM174, LRRC19 and SAA1 in the training cohort.

First, we conducted univariate COX analysis on the 102 M0-related DEGs to determine their prognostic significance (Supplementary Dataset File 2). Among these, 11 genes were identified and 10 were randomly selected for visualization (Fig. 4A). Our findings revealed that SAA1 and PPP1R1A were associated with a poor overall survival, whereas the other eight genes were associated with a better overall survival (Fig. 4A). Subsequently, we performed a LASSO analysis to identify the three potential candidates with the smallest lambda values and used them to construct a model of M0-related risk (Fig. 4B,C). Third, we performed multivariate COX analysis on these three M0-associated DEGs and eventually constructed a risk model based on these genes (TMEM174, LRRC19, and SAA1) (Fig. 4D) (Supplementary Dataset File 3). Finally, we categorized the samples into two subgroups based on the expression level of the three risk genes, after which we conducted a KM prognostic analysis and generated a survivorship curve. The high and low expression subgroups of the three model genes showed significant differences in survival. Specifically, populations with high expression of TMEM174 and LRRC19 had a better prognosis than those with low expression (Fig. 4E,F), the SAA1 high expression group had worse survival than those with low expression (Fig. 4G).

### Constructing a nomogram from risk models and clinicopathological features

**Figure 5:**
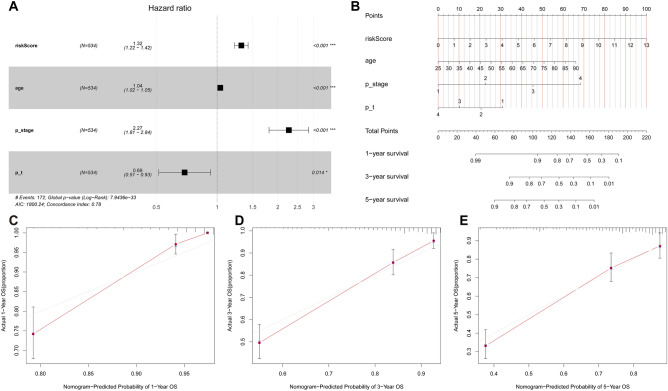
Constructing the nomogram. (**A**) A multivariate Cox regression analysis was conducted on the riskscore, age, sex, pathological stage, and previous malignancy. (**B**) A nomogram was constructed using risk score, age, sex, pathological stage and previous history of malignancy. (**C**–**E**) Calibration plot for nomogram in the test group.

To further analyze whether risk scores have an impact on the prognosis of ccRCC patients, we used univariate and multivariate regression analyses to determine whether risk scores are independent prognostic factors (Supplementary Dataset File 4 and 5). The findings revealed that risk score, age, and pathological stage were independent prognostic factors in ccRCC patients (Fig. 5A). Immediately following this, we integrated multiple metrics, including risk scores, age, and pathological staging and plotted nomograms to describe the intervariable correlations (Fig. 5B). The calibration curves showed that the nomogram with risk scores had high predictive power for patient survival at 1, 3, and 5 years (Fig. 5C–E).

### IRRM showed high prognostic value

**Figure 6:**
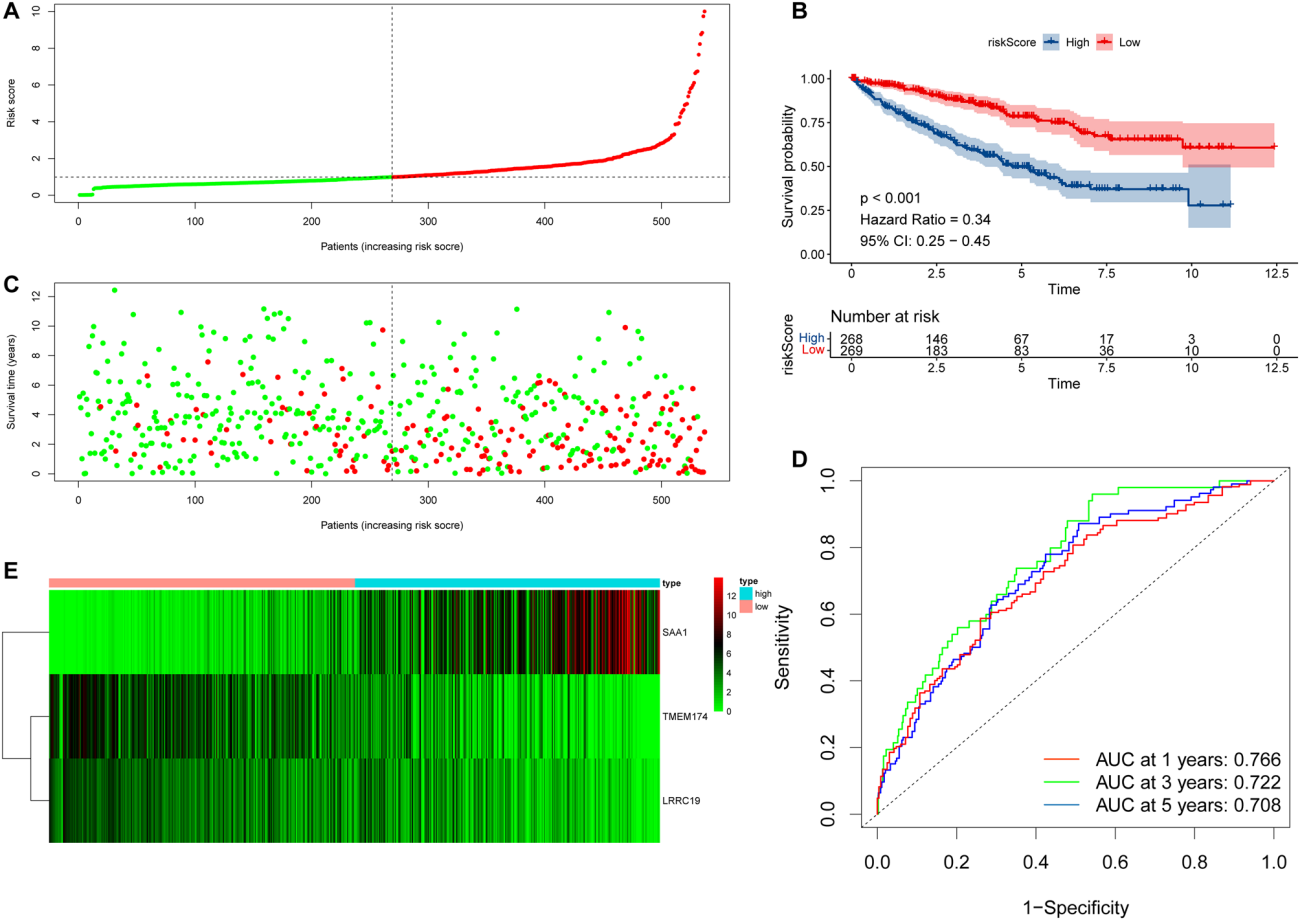
Risk models demonstrate high predictive value in training set. (**A**) Based on the results of the scoring, we divided the training cohort into a HRG and a LRG. (**B**) KM survival analysis results indicated differences between HRG and LRG. (**C**) The scatter plot results show surviving and dying cases in the HRG and LRG. (**D**) Time-dependent ROC analysis of immune risk models at 1, 3 and 5 years in the training cohort. (**E**) Expression of SAA1, TMEM174, LRRC19 in the HRG and LRG in the training cohort.

To further validate the predictive value of the risk model, we divided the ccRCC samples in the training cohort into the HRG and LRG based on the median risk score and conducted a prognostic analysis (Fig. 6A). Our findings revealed that the HRG had a worse overall survival rate (Fig. 6B) and a correspondingly higher mortality rate (Fig. 6C) than the LRG. Furthermore, we assessed the prognostic ability of IRRM using ROC curves, which showed that the sensitivity of IRRM in predicting overall survival at 1, 3, and 5 years was 0.766, 0.722, and 0.708, respectively (Fig. 6D). Furthermore, we observed different trends for the three model genes, with downregulated TMEM174 and LRRC19 expression downregulated and SAA1 expression upregulated in the HRG (Fig. 6E). These results demonstrate the excellent prognostic predictive value of risk models based on M0 in RCC.

### Risk score correlated with tumor stage and grade in the validation cohort

**Figure 7:**
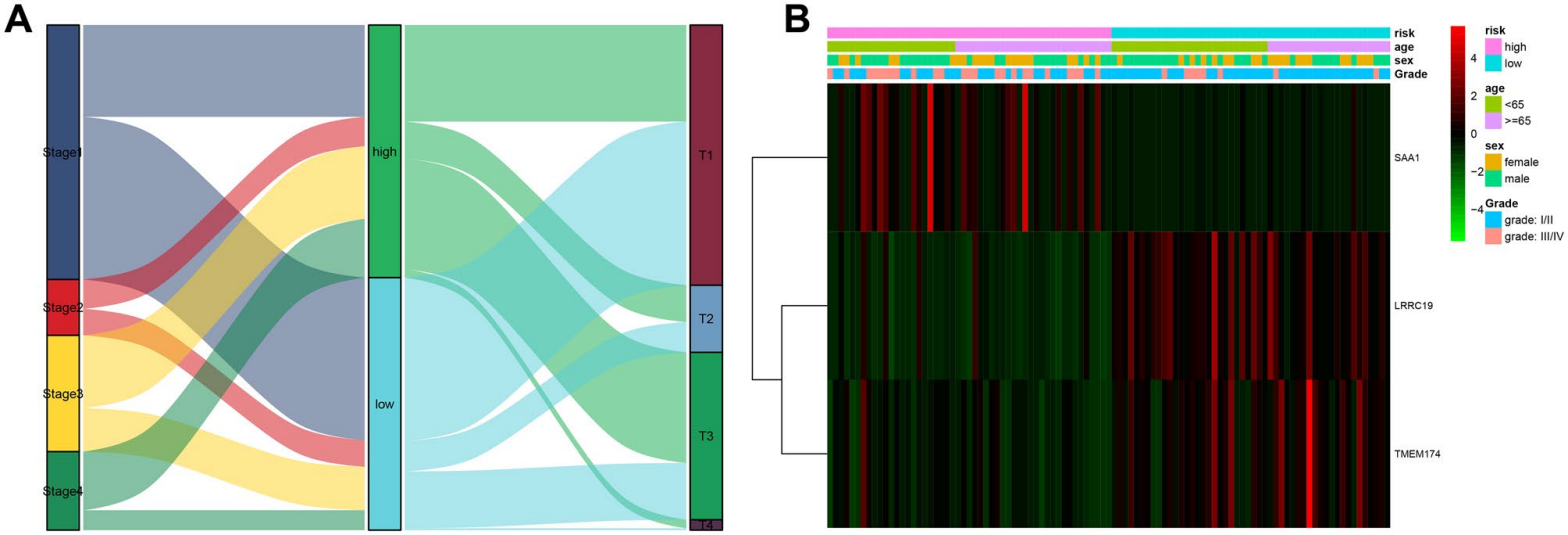
Riskscore correlated with ccRCC tumour stage and grade in the validation cohort. (**A**) Sankey demonstrates the relationship between risk score and tumour staging and grading in the validation cohort. (**B**) Clinical relevance of heat maps in relation to risk scores and tumour grading in a training cohort.

We rechecked the stability of the IRRM using another dataset (GSE40435). A Sankey diagram was created to examine the relationship between the risk score and various stages of tumor development. Our results indicated that the majority of stages 3/4 and T3/4 corresponded to the HRG (Fig. 7A). Similarly, grades III/IV were markedly more common in the HRG than in the LRG, whereas the reverse was true for grades I/II (Fig. 7B). However, there were no apparent differences in age or sex between the HRG and LRG (Fig. 7B). These findings suggest that the HRG had a poorer prognosis, which is the same as the results of the IRRM analyzed in the previous section. Furthermore, the expression trends of the three genes in the validation set were consistent with those in the training set. Specifically, TMEM174 and LRRC19 expression were downregulated in the HRG, whereas the expression of SAA1 was upregulated (Fig. 7B). In addition, another validation cohort was used to verify the robustness of the constructed model, and the samples of the validation cohort were divided into the HRG and LRG (Fig. S4A). The results showed that the HRG was associated with a worse prognosis (Fig. S4B), and higher mortality (Fig. S4C) compared with the LRG in the validation cohort. The ROC curve showed that the sensitivities of IRRM in predicting 1, 3, and 5 years overall survival were 0.611, 0.601, and 0.591, respectively (Fig. S4D). TMEM174 and LRRC19 were downregulated and SAA1 was upregulated in the HRG (Fig. S4E). In the validation cohort, KM survival results showed that the low TMEM174 and LRRC19 expression group had a lower survival probability than the high expression group (Fig. S4F,G), while the SAA1 low expression group had a significantly higher survival probability than the high expression group (Fig. S4H).

### Risk models related to immunity and the cancer-immunity cycle

**Figure 8:**
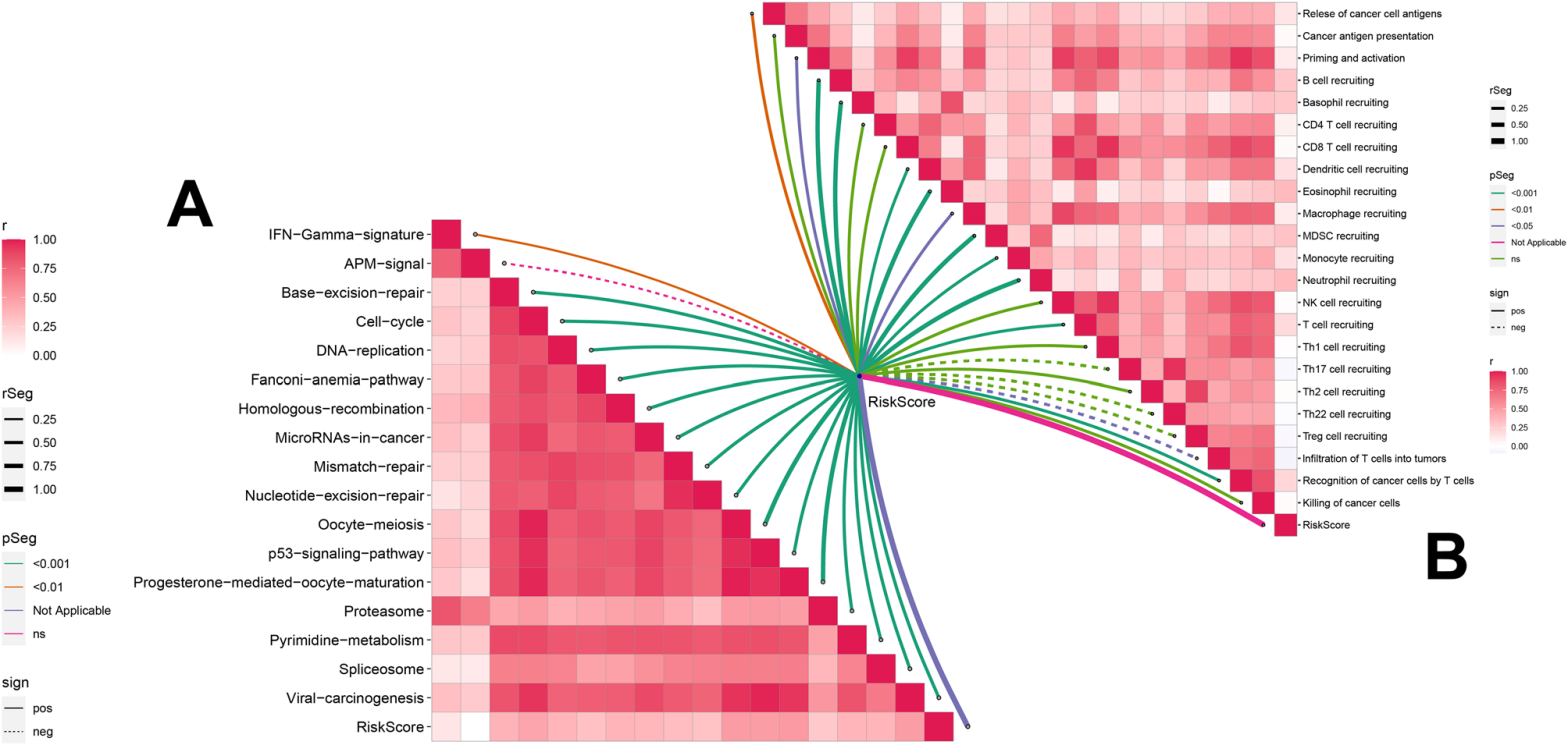
The immune risk model predicts the effect of immunotherapy and the cancer immune cycle. (**A**) The immune risk score correlates with the enrichment score of the immunotherapy prediction pathway. (**B**) Immunological risk scores are associated with the cancer-immunity cycle process.

We then demonstrated the potential link between the risk score and the cancer immunity cycle. Our findings revealed that the risk score exhibited a positive correlation with the GSVA enrichment score for all positive signals related to immunotherapy, except for the APM signal, suggesting that ccRCC may be closely associated with immunity and respond to immunotherapy (Fig. 8A).

Furthermore, we observed that the risk score was inversely linked with some functions of the tumor immunity cycle, such as th17 cell recruiting, th22 cell recruiting, Treg cell recruitment, and infiltration of T-cells into the tumor, while it was positively correlated with most of the steps of the cancer-immune cycle, including the release of cancer cell antigens, macrophages, MDSC, neutrophils, B cells, basophil cells, CD8+ T, NK, Th1, and Th2 cell recruitment (Fig. 8B).

### Risk models related to immunotherapy

**Figure 9:**
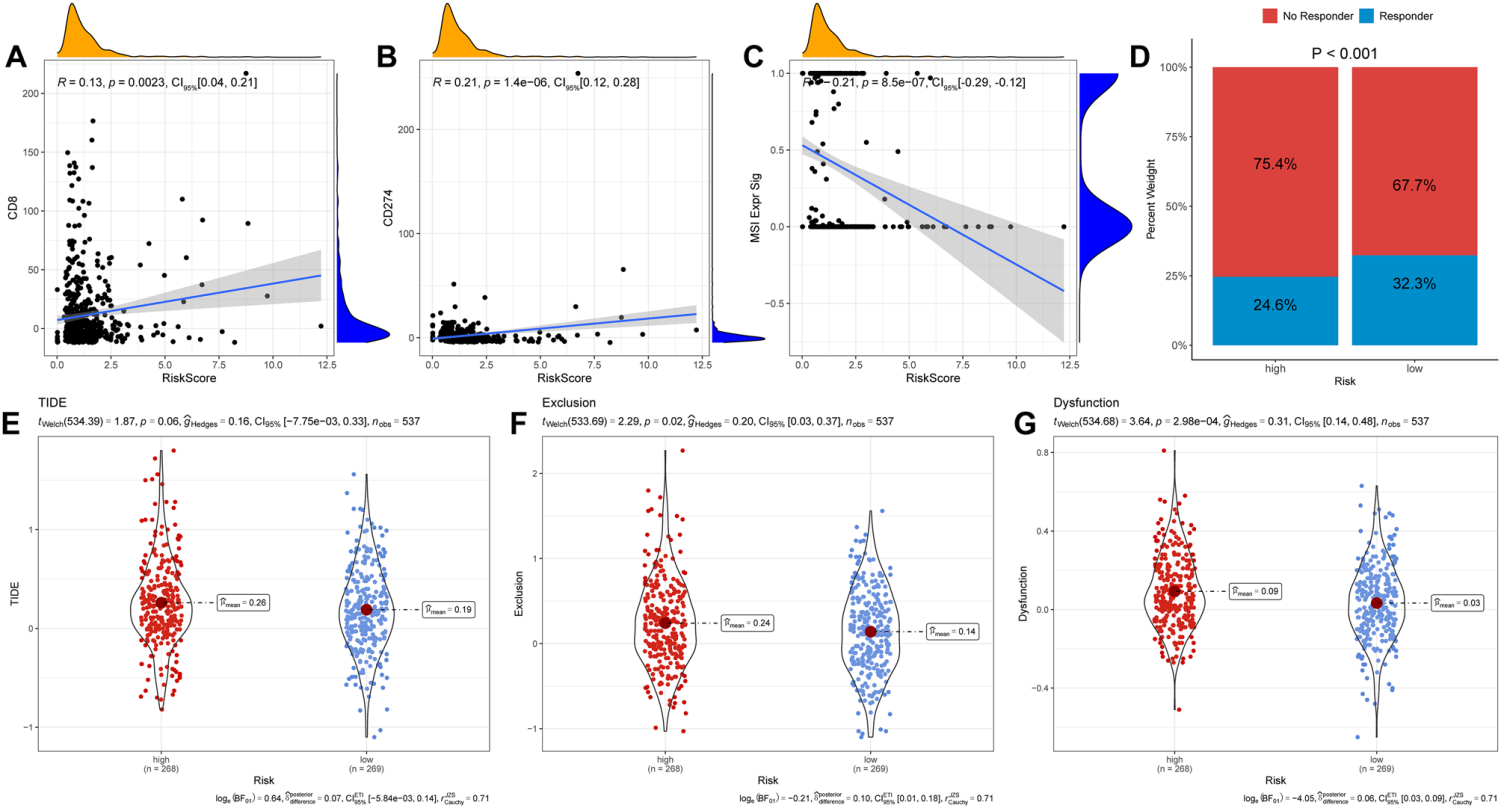
Samples from the HRG of ccRCC were more sensitive to ICIs treatment. (**A**–**C**) Correlation between risk scores and CD8, CD274 and MSI scores. (**D**) Prediction of sample sizes of non-responders and responders to ICIs treatment in HRG and LRG. (**E**–**G**) Differences in TIDE scores, Exclusion scores and Dysfunction scores between the HRG and LRG.

To investigate the association between the risk score and immunotherapy, we examined the immunological features of the two groups using TIDE. Our results indicated that the risk score increased with an increase in the expression of CD8 (Fig. 9A) and CD274 (Fig. 9B); however, the trend was opposite to that of MSI (Fig. 9C). Additionally, we performed TIDE analyses and found that exclusion, TIDE scores, and dysfunction differed between the two groups, with higher scores observed in the HRG (Fig. 9E–G). These findings suggest that the HRG of patients with ccRCC may have a strong immune escape, leading to poorer ICIs treatment outcomes. Furthermore, consistent with these findings, the percentage of individuals who did not respond to ICIs treatment was higher in the HRG (75.4%) than in the LRG (67.7%) (Fig. 9D).

### Relevance of risk models to IC

**Figure 10:**
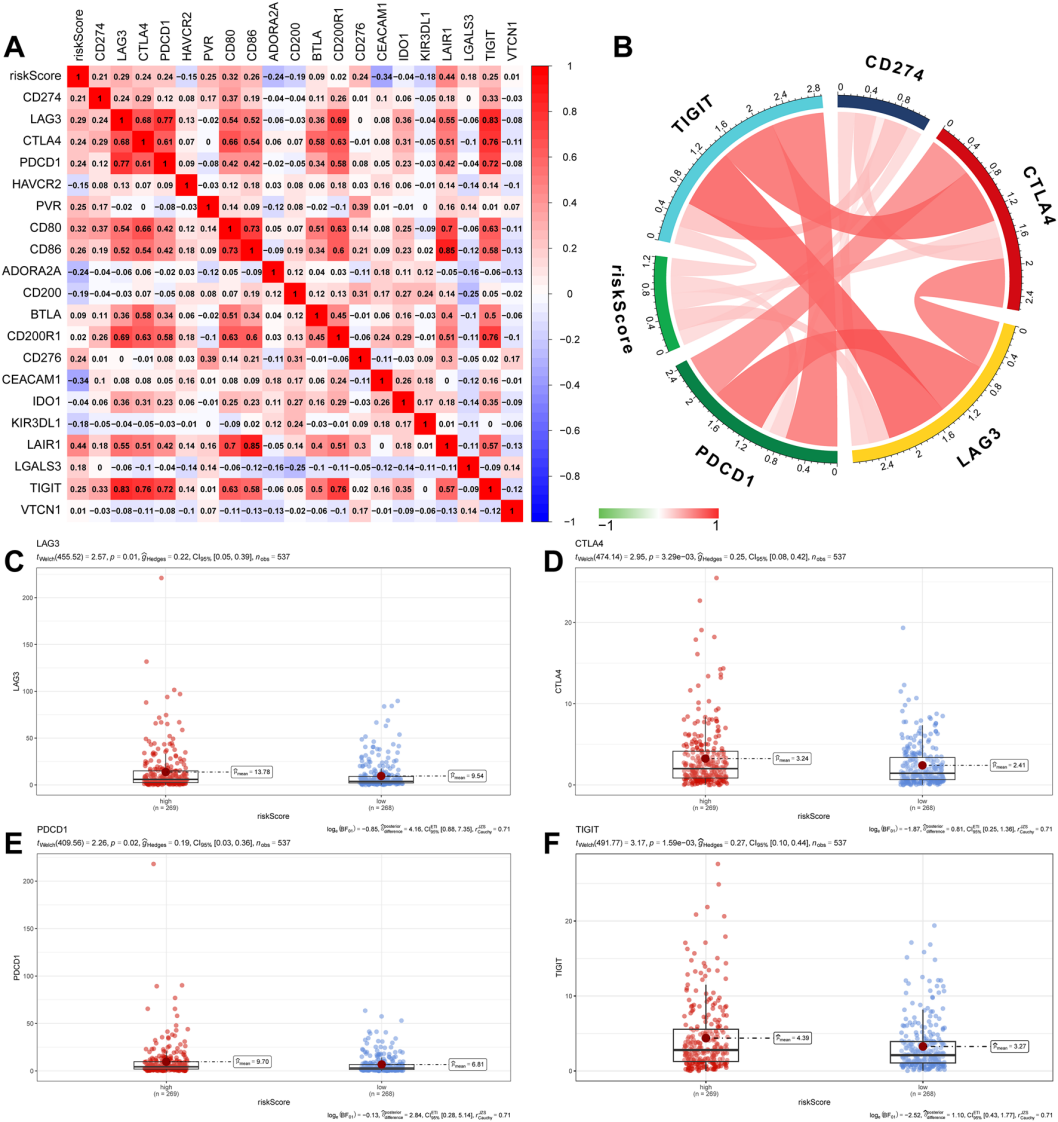
The relationship between riskscore and ICs. (**A**) Correlation between immune risk scores and ICs. (**B**) The correlations between immune-risk score and common ICs. (**C**–**F**) Differences in the expression of LAG3, CTLA4, PDCD1 and TIGIT genes in the HRG and LRG.

Next, we investigated the relationship between IRRM and ICs. Our findings revealed that the risk score was positively correlated with most ICs, including TIGIT, CTLA4, LAG3, CD274, and PDCD1 (Fig. 10A,B). Moreover, we compared the expression levels of LAG3, CTLA4, PDCD1, and TIGIT between the LRG and HRG. The expression of these ICs was remarkably higher in HRG than in LRG, as observed (Fig. 10C–F).

### Risk models relevant to targeted therapy

**Figure 11:**
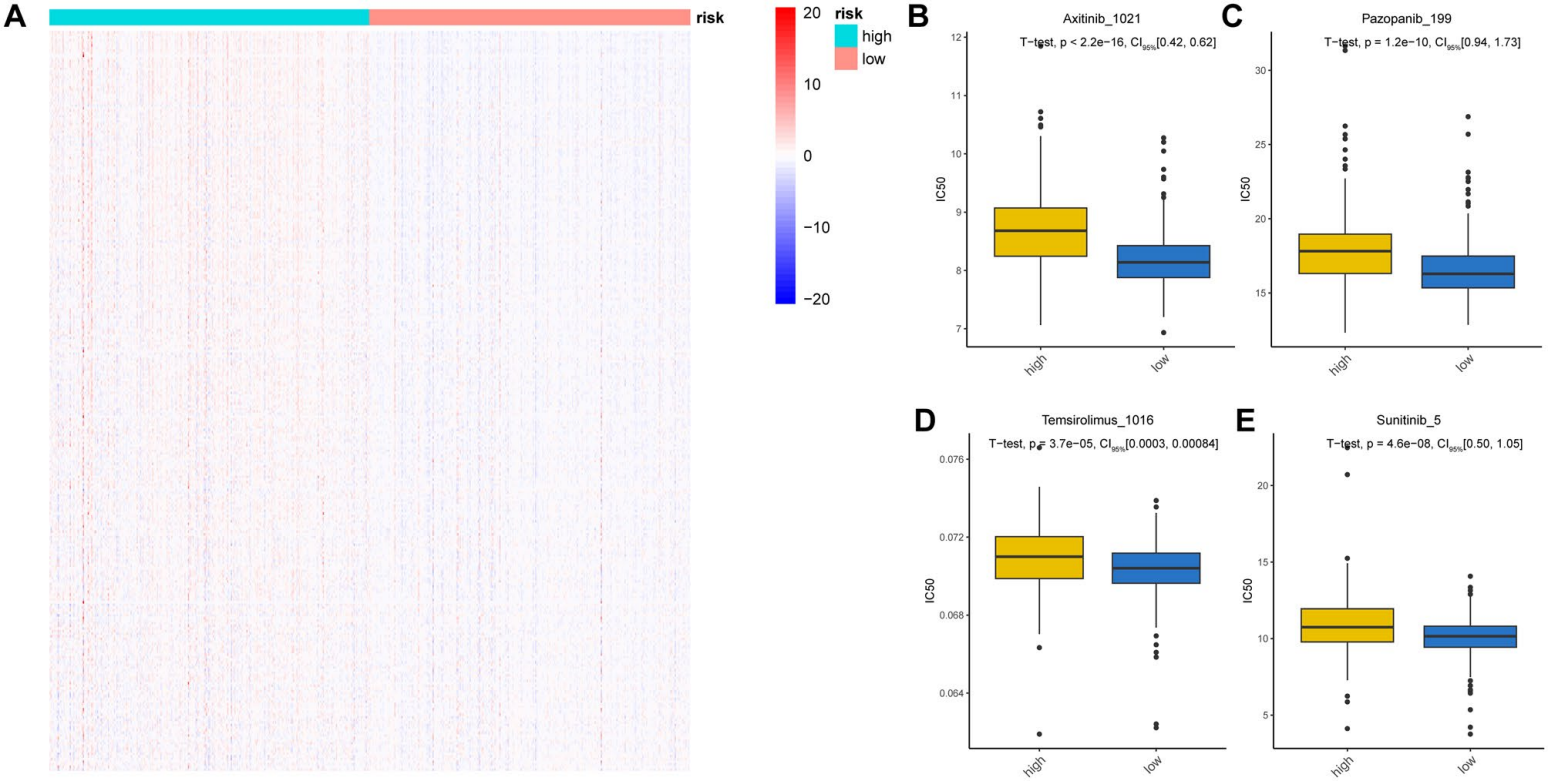
Targeted therapy for LRG and HRG. (**A**) Heat map plots show the differences in drug sensitivity between the HRG and LRG. (**B**–**E**) The box plot shows the difference in IC 50 between Axitinib, Pazopanib, Temsirolimus and Sunitinib in the HRG and LRG.

To explore the sensitivity of HRG and LRG patients to targeted drugs, we used the Oncopredict algorithm to convert the sensitivity scores of 349 drugs based on the gene expression matrix in ccRCC. Supplementary Dataset File 6 shows the scores for each sample. Our analyses revealed a substantial difference in sensitivity to 283 drugs between the HRG and LRG (Fig. 11A), with the HRG being more resistant to 255 drugs (Fig. 11A). Moreover, we identified 28 drugs, including Axitinib, Pazopanib, Temsirolimus, and Sunitinib, with lower IC50 values in the HRG than in the LRG (Fig. 11B–E).

### Immunohistochemical detection of SAA1 expression

**Figure 12:**
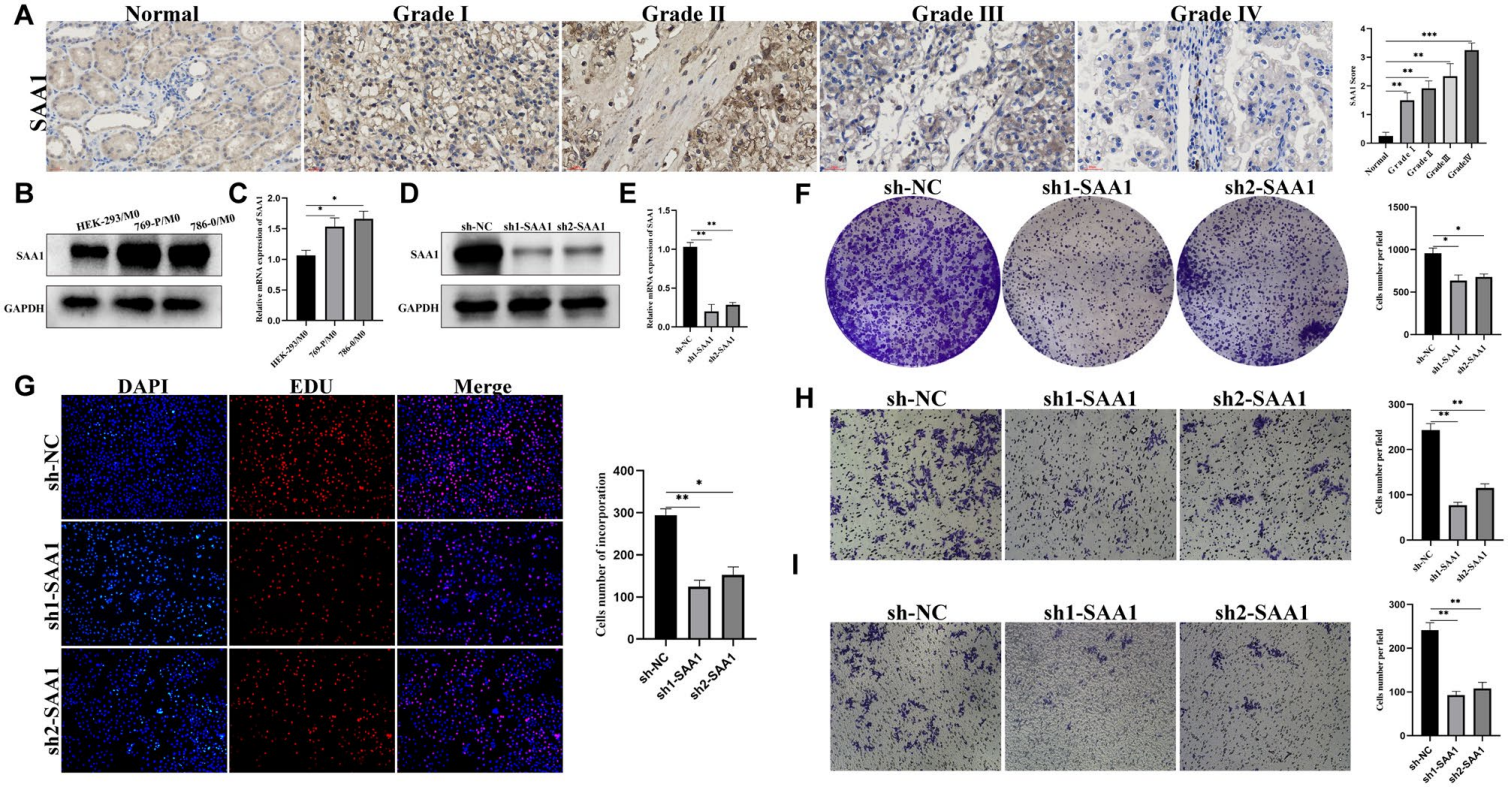
Functional validation of model genes. (**A**) Immunohistochemical results of SAA1 in tissues. (**B**,**C**) Differential expression of SAA1 in normal and tumour cells. (**D**,**E**) Changes in SAA1 protein and RNA levels in cells after SAA1 knockdown. (**F**,**G**) Knockdown of SAA1 reduced the proliferative capacity of the cells. (**H**,**I**) After knocking down SAA1, cell migration and invasion abilities were decreased. Due to performing multiple sets of experiments on a single blot, the full-length blots had to be cropped. However, we provide the results of three replicate experiments in Supplementary Figs. 5, 6 and 7. SAA1 and GAPDH were probed on the same gel after cutting into half.

Immunohistochemical staining demonstrated that SAA1 was absent in normal tissues but moderately or severely expressed in the immune cells of grade I, II, III, and IV tumor tissues and was located in the nucleus and cytoplasm (Fig. 12A). SAA1 was specifically expressed in RCC tissues and was significantly correlated with metastasis (p = 0.007), tumor stage (p = 0.022), and tumor grade (p = 0.015) (Table 1).

**Table 1:** Correlation between clinicopathological and SAA1 expression.

Parameters	n	SAA1 expression				p
		0	+ 1	+ 2	+ 3	
Age						
≤ Mean	39	2	18	13	6	0.822
≥ Mean	27	2	13	7	5	
Gender						
Male	41	3	20	11	7	0.584
Female	25	1	11	9	4	
Location						
Upper-pole	38	3	17	12	6	0.747
Middle	9	0	4	3	2	
Lower-pole	19	1	10	5	3	
Size						
< 40 mm	20	2	10	5	3	0.442
40–70 mm	31	2	15	9	5	
> 70 mm	15	0	6	6	3	
Metastasis						
Metastasis	21	1	6	7	7	0.015
Non-metastasis	45	3	25	13	4	
T-stage^a^						
T1	25	2	15	7	1	0.005
T2	19	1	12	4	2	
T3	11	1	2	4	4	
T4	11	0	2	5	4	
Grade^b^						
Well differentiated	29	2	18	6	3	0.018
Moderately differentiated	21	2	9	7	3	
Poorly differentiated	16	0	4	7	5	

^a^Tumor stage was determined using the 2009 TNM staging classification system.

^b^Tumor grade was determined using the Fuhrman classification system (well differentiated = grade 1 and 2, moderately differentiated = grade 3, and poorly differentiated = grade 4).

### SAA1 promotes cell proliferation

To verify whether macrophages can induce SAA1 expression in renal tumor cells. Co-incubation of renal tumor cells with macrophage medium promoted SAA1 expression in renal tumor cells (Fig. 12B,C). First knock down SAA1 expression in macrophages (Fig. 12D,E). Tumor cells were treated with control and knockdown SAA1 macrophage medium, and it was found that the knockdown of SAA1 macrophage medium significantly reduced the ability of tumor cells to proliferate, migrate, and invade (Fig. 12F–I), suggesting that SAA1 plays a role in promoting tumor growth.

## Discussion

The formation of an immunosuppressive TME is an indicator of tumor progression^40^. In this environment, tumor cells evade the immune system, which is a necessary precondition for tumor progression. Additionally, tumor cells must interconnect with other cells in the TME to provide the necessary signaling molecules and pathways. Macrophages are found in almost all body tissues, are highly flexible and differentiate into M1 and M2 under different conditions^41^. M0 macrophages, which are derived from the bone marrow, are generally believed to be precursors for M1 and M2 cells and are only in a quiescent state^42^. M1-like macrophages primarily eliminate pathogens and tumor cells, whereas M2-like macrophages are tumor-associated macrophages that play crucial roles in tumor development and immunosuppression^43^. Studies have indicated that Malignant gliomas are rich in M0 macrophages in addition to M1 and M2 cells that exhibit polarization^44^. It has even been suggested that M0 macrophages can be used to characterize glioblastoma malignancy^44^. Additionally, Liu et al. found that M0 macrophage infiltration was significantly higher in endometrial cancer^45^. In patients with high-grade tumors, M0 macrophages are associated with poor prognosis, indicating a role for M0 macrophages in promoting tumor growth^42^. Additionally, relevant studies have shown that elevated M0 cell levels in patients with early stage lung adenocarcinoma are correlated with reduced survival and poor patient prognosis^46^. Our results are consistent with these findings, as we showed that M0 macrophages are abundant in ccRCC tissues and are strongly associated with patient prognosis. Our analysis based on M0 macrophages showed that patients with a low abundance had a better prognosis than those with a high abundance.

SAA1 is an acute-phase reactive protein produced mainly in the liver that increases in response to injury and infection^47^. Elevated SAA1 levels are associated with many pathological conditions, including atherosclerosis and amyloidosis^48,49^. As an apolipoprotein, SAA regulates cholesterol metabolism by modulating lecithin/cholesterol acyltransferase activity or by altering the affinity pathway of high-density lipoprotein (HDL) for macrophages^50,51^. SAA1 also plays an important role in the immune system and serves as a diagnostic and prognostic marker for several diseases^52^. In glioblastoma and ovarian cancer, SAA1 shows promise as a potential therapeutic target and prognostic marker linked to immune infiltration^53,54^. Additionally, recent research on SAA1 has found that hypermethylation of a part of its promoter region is responsible for the significant upregulation of both transcription and protein expression in advanced RCC, indicating its diagnostic and prognostic value^55^. LRRC19 is a transmembrane receptor of the leucine-rich repeat (LRR) family that regulates the recruitment of immune cells to intestinal inflammation via associated chemokines^56^. Chai et al. proposed that LRRC19 induces pro-inflammatory cytokine expression through NF-κB activation and is involved in the inflammatory response^57^. In some cancers, such as ovarian, gastric, breast, lung, and colorectal cancers, a significant association between the downregulation of LRRC19 expression and poor patient prognosis has been observed^58^. According to the relevant literature, LRRC19 serves as both a prognostic indicator in RCC and a potentially useful prognostic and adjuvant therapeutic marker in ccRCC^59^. TMEM174, a recently discovered protein, consists of 243 amino acids and is highly expressed in human kidney tissues. It promotes cell proliferation by activating AP-1 and regulates NaPi2a, which is considered a regulator that prevents hyperphosphatemia^60,61^. Additionally, TMEM174 is differentially expressed in various RCC subtypes, with high expression in squamous cell carcinoma and migratory cell carcinoma and low expression in ccRCC, retroperitoneal metastatic ccRCC, and adrenal metastatic ccRCC^62^. In our investigation, we used differential gene analysis, multivariate regression analysis, and LASSO analysis to construct risk models for SAA1, TMEM174, and LRRC19 in M0 cells with high and low infiltration. Survival analysis of the three genes revealed that high expression of TMEM174 and LRRC19 indicated good prognosis, indicating their role as protective genes, whereas high expression of SAA1 indicated poor prognosis, implying that they function as risk genes. These results are consistent with those of previous studies, further emphasizing the significant roles of these genes in ccRCC. Furthermore, we conducted multivariate regression and prognostic analyses on the model based on SAA1, TMEM174, and LRRC19 and discovered that the risk score was an independent factor for evaluating the prognosis of patients with ccRCC, with those in the HRG having a poorer prognosis. ROC analysis indicated an AUC greater than 0.7, implying that the risk model had good predictive efficacy for 1-, 3-, and 5-year overall survival.

Immunotherapy and targeted therapies have made significant progress in recent years and show great potential for treating a wide range of cancers. Owing to the low efficacy of radiotherapy and chemotherapy, these treatments have become important options for patients with RCC^63^. The VEGF signaling pathway is a key driver of tumor vascular development and its inhibition is one of the primary therapeutic approaches for advanced RCC^64^. For instance, sunitinib is known to inhibit VEGF and PDGF receptors and has shown considerable improvement in the progression-free survival of patients^65^. Furthermore, pazopanib inhibits tyrosine kinase, which targets VEGF receptors 1, 2, and 3, PDGF receptors, and other signaling pathways. In a Phase III trial of first-line treatment, pazopanib demonstrated similar effectiveness to sunitinib in improving progression-free survival^66^. Moreover, inhibition of the mTOR pathway can have a profound impact on the functions of renal cancer cells such as cell growth, proliferation, metabolism, and angiogenesis^67^. Sirolimus is a well-known inhibitor of the mTOR pathway, which is downstream of the PI3K and Akt pathways. A Phase III trial involving 626 previously untreated low-risk patients confirmed the effectiveness and safety of tisirolimus, with a significantly improved overall survival benefit in the tisirolimus group as compared to IFN-α^68^. Tumor cells evade the immune system by interacting with PD-1 and PD-L1, leading to the development of PD-1 or PD-L1 checkpoint inhibitors with promising efficacy^69^. Tremendous advances in ICIs therapy have opened new therapeutic prospects for patients with cancer, and ICIs are now extensively used in the treatment of various cancer types. The main immune checkpoints are PD-L1, PD-1, and CTLA-4. RCC is highly immunogenic, with approximately 30% of RCC cases showing PD-L1^70^. Opdivo is an immune checkpoint inhibitor antibody that is a humanized monoclonal immunoglobulin with a selective affinity for PD-1 expressing immune cells. This innovative therapy has shown promise and has been approved for the treatment of metastatic melanoma and non-small cell lung cancer^71^. Studies using Opdivo in treatment (CheckMate-025) have demonstrated significantly superior overall survival, hazard ratio, and objective response rate (ORR)^71^. However, it is important to note that although these treatments have improved the outcome for patients with RCC, relapse or progression of the disease still occurs occasionally, and targeted therapies and immunotherapies are less effective in a subset of patients. Consequently, identifying potential targets or immune-associated genes with significant therapeutic value is critical to improve the prognosis of patients with advanced RCC.

TIDE is a recently developed computational approach designed to predict a sample's response to ICIs treatment by combining two parameters: the T-cell dysfunction score and T-cell rejection score. However, in ccRCC, immune escape mainly occurs through T-cell dysfunction, resulting in higher T-cell levels and dysfunction scores^38^. Additionally, researchers have observed a negative correlation between T cell dysfunction and exclusion in five types of cancer^38^. Another important factor related to the efficacy of ICIs therapy is the MSI score^72^. In our study, we evaluated the correlation between risk scores and ICs, including TIGIT, CTLA-4, LAG3, PD-L1, and PDCD1, and found a positive correlation between them. In addition, we observed a negative correlation between MSI and risk scores.

Similar to previous findings, our study confirmed the presence of significant immune escape in the HRG, as evidenced by higher TIDE scores, exclusion, and T-cell dysfunction. Interestingly, we also found that the proportion of patients responding to ICIs treatment in the HRG was lower than that in the LRG, indicating that patients in the LRG may be more responsive to ICIs treatment and potentially benefit more from it. Finally, we performed sensitivity analyses of the targeted drugs in HRG and LRG patients using the OncoPredict algorithm. We calculated the sensitivity scores for 349 drugs and found that for 283 drugs, there was a difference in sensitivity between the HRG and LRG. The HRG exhibited the greatest resistance to 255 drugs. Meanwhile, the HRG was more sensitive to treatment with 28 drugs (axitinib, pazopanib, temsirolimus, and sunitinib) and had lower IC50 values than the LRG. In conclusion, our study suggests that although there is significant immune evasion in ccRCCs with HRG, their response to immunotherapy is not as favorable as that in ccRCCs with LRG. Moreover, their sensitivity to commonly targeted drugs is poor, leading to poor prognosis in patients with LRG.

As a risk gene, SAA1 may be closely associated with tumor formation and progression. Therefore, we verified the expression of SAA1 at the protein level using immunohistochemistry of the collected paraffin sections. Our results indicated that SAA1 was expressed only in tumor tissues and was associated with tumor metastasis, tumor T stage, and tumor grade, which is also consistent with our analysis. Furthermore, studies that interfere with SAA1 have suggested that SAA1 promotes the proliferation and invasion of renal tumor cells.

Unlike Fu et al.^73^, who developed a risk model for ccRCC based on genes related to necroptosis and pyroptosis, we developed a risk model based on genes related to tumor infiltration M0 macrophages. Fu et al.^73^ screened 10 genes related to necroptosis and pyroptosis to construct a risk model based on TCGA and GEO ccRCC datasets. Lin et al.^74^ used TGCA, ArrayExpress and checkmat-025 (CM-025) cohort datasets to analyze the characteristics of fatty acid metabolism-related mRNA, and screened 6 fatty acid metabolisM-related genes for the construction of risk models. Bao et al.^75^ screened 6 necroptosis related miRNAs from TCGA database to construct the risk model. In our study, we comprehensively used ccRCC datasets from TCGA, GEO and ArrayExpress databases to construct a risk model for related research. The AUC values of the risk model constructed by Fu et al.^73^ for predicting the 1-, 3-, and 5-year survival probability of ccRCC patients were 0.763, 0.707, and 0.728, respectively. The AUC values of the model constructed by Lin et al.^74^ for predicting the 3-, 5-, and 7-year survival probability of ccRCC patients were 0.745, 0.765, and 0.742, respectively. The AUC values of Bao et al.^75^ model in predicting the 1-, 3-, and 5-year survival probability of ccRCC patients were 0.721, 0.711, and 0.703, respectively. The AUC values of our model for predicting the 1-, 3-, and 5-year survival probability of ccRCC patients were 0.766, 0.722, and 0.708, respectively, which was similar to the prediction ability of Fu et al.'s model. Fu et al.^73^ showed that infiltrating M0 macrophages were positively correlated with the risk score, and the expression levels of model genes AIM2, CASP4, GSDMB, NOD2 and RBCK1 in tumor tissues were higher than those in adjacent normal tissues, among which CASP4 and GSDMB promoted the proliferation, migration and invasion of ccRCC cells. Lin et al.^74^ found that the expression levels of model genes ABCD1, ALOX12B, ALOX15B, CPT1B, and IL4I1 were up-regulated in tumor tissues, whereas HACD1 expression was lower in ccRCC tissues and the proportion of infiltrating M0 in high-risk group was higher than that in low-risk group. Furthermore, knockdown of IL4I1 in tumor cells inhibited the differentiation of M0 into M2 and inhibited the proliferation, migration and invasion of ccRCC cells. Bao et al.^75^ showed that the expression of risk model gene has-miR-193a-3p was significantly increased in ccRCC tissues, while the expression of miR-214-3p was significantly decreased. Our results showed that high M0 infiltration was associated with poor prognosis of ccRCC patients, and the expression of model gene SAA1 in tumor tissues was higher than that in normal tissues. Knockdown of SAA1 could inhibit the proliferation and migration of ccRCC tumor cells. Fu and Lin et al.^73,74^ also found that the infiltrating abundance of M0 was correlated with the survival prognosis of ccRCC patients. Fu et al.^73^ showed that ccRCC patients in high score group were sensitive to ICI, targeted drugs as well as rapamycin and temsirolimus, suggesting that these patients may achieve better results by receiving targeted therapy combined with immunotherapy. Lin et al.^74^ found that the risk score can predict the clinical efficacy of ICI-based immunotherapy, and ccRCC patients in the low-risk group are more sensitive to ICI. These findings are helpful for the screening of ccRCC therapeutic drugs and the precision treatment of ccRCC patients. Bao et al.^75^ showed that the target genes of miRNA TGFBR3, SIX4, DUSP1 and ARHGAP42 were positively correlated with drug sensitivity, while TAL1 and BTG2 were negatively correlated. The above results suggested that we can choose different therapeutic methods according to the expression level of related model genes in ccRCC patients in the future, and may achieve better results. For example, we can assess the prognosis of ccRCC patients by examining the abundance of M0 macrophage infiltration and the expression of model related genes in ccRCC tissues. For patients with high M0 infiltration or high expression of model genes, the prognosis may be poor, and intensive management combined with multiple combination therapies may improve the prognosis. In addition, more sensitive to targeted therapy in ccRCC patients with LRG, HSG patients may have a better response to immunotherapy. However, these need to be further confirmed in future studies.

In our study, the risk model built based on DEGs between the high-infiltration and low-infiltration M0 groups showed high prognostic value in both the training and validation datasets and was closely related to targeted therapy and immunotherapy of RCC. Therefore, in clinical practice, we can first assess the prognosis of RCC patients by detecting the infiltration abundance of M0 macrophages and the expression of related genes in the tumor tissues of patients. For the group with high infiltration of M0, the prognosis may be poor; therefore, we can improve the prognosis by strengthening management combined with multiple treatment combinations. According to our findings, patients in the LSG were more sensitive to targeted therapies, including Axitinib, Pazopanib, Temsirolimus and Sunitinib. Patients with HSG have a poorer prognosis and may need to respond better to immunotherapy. However, relevant large-sample multi-center verification and more basic research is needed to clarify the specific mechanism by which M0 affects ccRCC for our study. Moreover, M0 key genes may be transformed into new RCC tumor biomarkers for the diagnosis and prognosis of RCC in the future. For example, M0 macrophage-associated SAA1 is a potential new molecular target for RCC therapy. In the future, the relationship between infiltrating immune cells in the tumor microenvironment and tumor progression will gradually attract attention. M0 macrophages can differentiate into M1 or M2 cells when stimulated by different tumor microenvironments and signals. Further analysis of the relationship between M0, M1, and M2 macrophages in ccRCC is required. Owing to the differences in infiltrating immune cells in the tumor microenvironment between individuals, it is of great significance to guide clinical treatment and prognosis according to the differences in immune cell infiltration while grasping the common characteristics of the tumor microenvironment between individuals.

In conclusion, the risk model constructed using M0 macrophage-associated genes in the ccRCC tumor microenvironment can predict the prognosis of patients with CCRCC and the effect of immunotherapy. In addition, we found that the model gene SAA1 possessed the ability promoted clear renal cell proliferation and migration. This discovery may offer novel insights and avenues for prognostic prediction and therapeutic approaches for RCC. However, we must also clearly recognize the shortcomings in our work. First, the data used in this study were obtained from public databases, which may suffer from data bias and insufficient sample size. Second, the results of this study need to be validated in different cohorts and large samples. Finally, this study only verified the functional role of the genes at the cellular level, and the results need to be further verified by in vivo experiments.

### Supplementary Information

Supplementary Information 1.

Supplementary Information 2.

Supplementary Information 3.

Supplementary Information 4.

Supplementary Information 5.

Supplementary Information 6.

Supplementary Figure 1.

Supplementary Figure 2.

Supplementary Figure 3.

Supplementary Figure 4.

Supplementary Figure 5.

Supplementary Figure 6.

Supplementary Figure 7.

## Data Availability

The ccRCC sample data used were from The Cancer Genome Atlas (TCGA) (https://portal.gdc.cancer.gov/analysis_page?app=Downloads), the Gene Expression Omnibus (GEO) database (cohort number: GSE40435) (https://www.ncbi.nlm.nih.gov/geo/query/acc.cgi?acc=GSE40435) and ArrayExpress database (E-MTAB-1980) (https://www.ebi.ac.uk/biostudies/arrayexpress/studies?query=E-MTAB-1980). The data used to support the findings of this study are available on request from the corresponding authors.

## References

[1] Ferlay J, Soerjomataram I, Dikshit R, Eser S, Mathers C, Rebelo M, Parkin DM, Forman D, Bray F (2015). Cancer incidence and mortality worldwide: Sources, methods and major patterns in GLOBOCAN 2012. Int. J. Cancer.

[2] Bray F, Ferlay J, Soerjomataram I, Siegel RL, Torre LA, Jemal A (2018). Global cancer statistics 2018: GLOBOCAN estimates of incidence and mortality worldwide for 36 cancers in 185 countries. CA Cancer J. Clin..

[3] Chen W, Zheng R, Baade PD, Zhang S, Zeng H, Bray F, Jemal A, Yu XQ, He J (2016). Cancer statistics in China, 2015. CA Cancer J. Clin..

[4] Saad OA, Li WT, Krishnan AR, Nguyen GC, Lopez JP, McKay RR, Wang-Rodriguez J, Ongkeko WM (2022). The renal clear cell carcinoma immune landscape. Neoplasia.

[5] Sun Z, Tao W, Guo X, Jing C, Zhang M, Wang Z, Kong F, Suo N, Jiang S, Wang H (2022). Construction of a lactate-related prognostic signature for predicting prognosis, tumor microenvironment, and immune response in kidney renal clear cell carcinoma. Front. Immunol..

[6] Zhou QH, Li KW, Chen X, He HX, Peng SM, Peng SR, Wang Q, Li ZA, Tao YR, Cai WL, Liu RY, Huang H (2020). HHLA2 and PD-L1 co-expression predicts poor prognosis in patients with clear cell renal cell carcinoma. J. Immunother. Cancer.

[7] Ruan B, Feng X, Chen X, Dong Z, Wang Q, Xu K, Tian J, Liu J, Chen Z, Shi W, Wang M, Qian L, Ding Q (2020). Identification of a set of genes improving survival prediction in kidney renal clear cell carcinoma through integrative reanalysis of transcriptomic data. Dis. Markers.

[8] Checkpoint inhibitor-TKI combos effective in RCC. *Cancer**Discov*. **9**(4), 460. 10.1158/2159-8290.CD-NB2019-024 (2019). 10.1158/2159-8290.CD-NB2019-024 30787015

[9] Choueiri TK, Escudier B, Powles T (2016). Cabozantinib versus everolimus in advanced renal cell carcinoma (METEOR): Final results from a randomised, open-label, phase 3 trial. Lancet Oncol..

[10] Rizzo A, Mollica V, Tateo V (2023). Hypertransaminasemia in cancer patients receiving immunotherapy and immune-based combinations: The MOUSEION-05 study. Cancer Immunol. Immunother..

[11] Rosellini M, Marchetti A, Mollica V, Rizzo A, Santoni M, Massari F (2023). Prognostic and predictive biomarkers for immunotherapy in advanced renal cell carcinoma. Nat. Rev. Urol..

[12] Guven DC, Sahin TK, Erul E (2022). The association between albumin levels and survival in patients treated with immune checkpoint inhibitors: A systematic review and meta-analysis. Front. Mol. Biosci..

[13] Rizzo A, Mollica V, Dall'Olio FG (2021). Quality of life assessment in renal cell carcinoma Phase II and III clinical trials published between 2010 and 2020: A systematic review. Future Oncol..

[14] Li M, Zha X, Wang S (2021). The role of N6-methyladenosine mRNA in the tumor microenvironment. Biochim. Biophys. Acta Rev. Cancer.

[15] Lei X, Lei Y, Li JK, Du WX, Li RG, Yang J, Li J, Li F, Tan HB (2020). Immune cells within the tumor microenvironment: Biological functions and roles in cancer immunotherapy. Cancer Lett..

[16] Lambert AW, Pattabiraman DR, Weinberg RA (2017). Emerging biological principles of metastasis. Cell.

[17] Celià-Terrassa T, Kang Y (2018). Metastatic niche functions and therapeutic opportunities. Nat. Cell Biol..

[18] Faubert B, Solmonson A, DeBerardinis RJ (2020). Metabolic reprogramming and cancer progression. Science.

[19] González-Tablas Pimenta M, Otero Á, Arandia Guzman DA, Pascual-Argente D, Ruíz Martín L, Sousa-Casasnovas P, García-Martin A, Roa Montes de Oca JC, Villaseñor-Ledezma J, Torres Carretero L, Almeida M, Ortiz J, Nieto A, Orfao A, Tabernero MD (2021). Tumor cell and immune cell profiles in primary human glioblastoma: Impact on patient outcome. Brain Pathol..

[20] Jiang X, Shapiro DJ (2014). The immune system and inflammation in breast cancer. Mol. Cell. Endocrinol..

[21] Gasparoto TH, de Souza Malaspina TS, Benevides L, de Melo Jr EJ, Costa MR, Damante JH, Ikoma MR, Garlet GP, Cavassani KA, da Silva JS, Campanelli AP (2010). Patients with oral squamous cell carcinoma are characterized by increased frequency of suppressive regulatory T cells in the blood and tumor microenvironment. Cancer Immunol. Immunother..

[22] Sungur CM, Murphy WJ (2014). Positive and negative regulation by NK cells in cancer. Crit. Rev. Oncog..

[23] Shang S, Yang YW, Chen F, Yu L, Shen SH, Li K, Cui B, Lv XX, Zhang C, Yang C, Liu J, Yu JJ, Zhang XW, Li PP, Zhu ST, Zhang HZ, Hua F (2022). TRIB3 reduces CD8^+^ T cell infiltration and induces immune evasion by repressing the STAT1-CXCL10 axis in colorectal cancer. Sci. Transl. Med..

[24] Liu Z, Wang T, She Y, Wu K, Gu S, Li L, Dong C, Chen C, Zhou Y (2021). N^6^-methyladenosine-modified circIGF2BP3 inhibits CD8^+^ T-cell responses to facilitate tumor immune evasion by promoting the deubiquitination of PD-L1 in non-small cell lung cancer. Mol. Cancer.

[25] Fang W, Zhou T, Shi H, Yao M, Zhang D, Qian H, Zeng Q, Wang Y, Jin F, Chai C, Chen T (2021). Progranulin induces immune escape in breast cancer via up-regulating PD-L1 expression on tumor-associated macrophages (TAMs) and promoting CD8^+^ T cell exclusion. J. Exp. Clin. Cancer Res..

[26] Zhou K, Cheng T, Zhan J, Peng X, Zhang Y, Wen J, Chen X, Ying M (2020). Targeting tumor-associated macrophages in the tumor microenvironment. Oncol. Lett..

[27] Fu C, Jiang A (2018). Dendritic cells and CD8 T cell immunity in tumor microenvironment. Front. Immunol..

[28] Xie Y, Chen Z, Zhong Q (2021). M2 macrophages secrete CXCL13 to promote renal cell carcinoma migration, invasion, and EMT. Cancer Cell Int..

[29] Gabrusiewicz K, Rodriguez B, Wei J (2016). Glioblastoma-infiltrated innate immune cells resemble M0 macrophage phenotype. JCI Insight.

[30] Huang L, Wang Z, Chang Y (2020). EFEMP2 indicates assembly of M0 macrophage and more malignant phenotypes of glioma. Aging (Albany NY).

[31] Newman AM, Liu CL, Green MR (2015). Robust enumeration of cell subsets from tissue expression profiles. Nat. Methods.

[32] Kanehisa M, Goto S (2000). KEGG: Kyoto encyclopedia of genes and genomes. Nucleic Acids Res..

[33] Kanehisa M (2019). Toward understanding the origin and evolution of cellular organisms. Protein Sci..

[34] Kanehisa M, Furumichi M, Sato Y, Kawashima M, Ishiguro-Watanabe M (2023). KEGG for taxonomy-based analysis of pathways and genomes. Nucleic Acids Res..

[35] Park SY (2018). Nomogram: An analogue tool to deliver digital knowledge. J. Thorac. Cardiovasc. Surg..

[36] Hu J, Yu A, Othmane B, Qiu D, Li H, Li C, Liu P, Ren W, Chen M, Gong G, Guo X, Zhang H, Chen J, Zu X (2021). Siglec15 shapes a non-inflamed tumor microenvironment and predicts the molecular subtype in bladder cancer. Theranostics.

[37] Chen DS, Mellman I (2013). Oncology meets immunology: The cancer-immunity cycle. Immunity.

[38] Jiang P, Gu S, Pan D, Fu J, Sahu A, Hu X, Li Z, Traugh N, Bu X, Li B, Liu J, Freeman GJ, Brown MA, Wucherpfennig KW, Liu XS (2018). Signatures of T cell dysfunction and exclusion predict cancer immunotherapy response. Nat. Med..

[39] Maeser D, Gruener RF, Huang RS (2021). oncoPredict: An R package for predicting in vivo or cancer patient drug response and biomarkers from cell line screening data. Brief Bioinform..

[40] Allegrezza MJ, Conejo-Garcia JR (2017). Targeted therapy and immunosuppression in the tumor microenvironment. Trends Cancer.

[41] Salmaninejad A, Valilou SF, Soltani A, Ahmadi S, Abarghan YJ, Rosengren RJ, Sahebkar A (2019). Tumor-associated macrophages: Role in cancer development and therapeutic implications. Cell Oncol. (Dordr.).

[42] Zhang Y, Zou J, Chen R (2022). An M0 macrophage-related prognostic model for hepatocellular carcinoma. BMC Cancer.

[43] Pucci M, Raimondo S, Urzì O, Moschetti M, Di Bella MA, Conigliaro A, Caccamo N, La Manna MP, Fontana S, Alessandro R (2021). Tumor-derived small extracellular vesicles induce pro-inflammatory cytokine expression and pd-l1 regulation in M0 macrophages via IL-6/STAT3 and TLR4 signaling pathways. Int. J. Mol. Sci..

[44] Huang L, Wang Z, Chang Y, Wang K, Kang X, Huang R, Zhang Y, Chen J, Zeng F, Wu F, Zhao Z, Li G, Huang H, Jiang T, Hu H (2020). *EFEMP2* indicates assembly of M0 macrophage and more malignant phenotypes of glioma. Aging (Albany NY).

[45] Liu J, Chen X, Jiang Y, Cheng W (2020). Development of an immune gene prognostic classifier for survival prediction and respond to immunocheckpoint inhibitor therapy/chemotherapy in endometrial cancer. Int. Immunopharmacol..

[46] Liu X, Wu S, Yang Y, Zhao M, Zhu G, Hou Z (2017). The prognostic landscape of tumor-infiltrating immune cell and immunomodulators in lung cancer. Biomed. Pharmacother..

[47] Cao K, Jiang X, Wang B, Ni Z, Chen Y (2022). SAA1 expression as a potential prognostic marker of the tumor microenvironment in glioblastoma. Front. Neurol..

[48] Getz GS, Krishack PA, Reardon CA (2016). Serum amyloid A and atherosclerosis. Curr. Opin. Lipidol..

[49] Villapol S, Kryndushkin D, Balarezo MG, Campbell AM, Saavedra JM, Shewmaker FP, Symes AJ (2015). Hepatic expression of serum amyloid A1 is induced by traumatic brain injury and modulated by telmisartan. Am. J. Pathol..

[50] Yamada T, Wada A, Itoh K, Igari J (2000). Serum amyloid A secretion from monocytic leukaemia cell line THP-1 and cultured human peripheral monocytes. Scand. J. Immunol..

[51] Cabana VG, Feng N, Reardon CA, Lukens J, Webb NR, de Beer FC, Getz GS (2004). Influence of apoA-I and apoE on the formation of serum amyloid A-containing lipoproteins in vivo and in vitro. J. Lipid Res..

[52] De Buck M, Gouwy M, Wang JM, Van Snick J, Proost P, Struyf S, Van Damme J (2016). The cytokine-serum amyloid A-chemokine network. Cytokine Growth Factor Rev..

[53] Cui G, Xiao Y (2022). Identification of SAA1 as a prognostic biomarker associated with immune infiltration in glioblastoma. Autoimmunity.

[54] Li Z, Hou Y, Zhao M, Li T, Liu Y, Chang J, Ren L (2020). Serum amyloid a, a potential biomarker both in serum and tissue, correlates with ovarian cancer progression. J. Ovarian Res..

[55] Li S, Cheng Y, Cheng G, Xu T, Ye Y, Miu Q, Cao Q, Yang X, Ruan H, Zhang X (2021). High SAA1 expression predicts advanced tumors in renal cancer. Front. Oncol..

[56] Cao S, Su X, Zeng B, Yan H, Huang Y, Wang E, Yun H, Zhang Y, Liu F, Li W, Wei H, Che Y, Yang R (2016). The gut epithelial receptor LRRC19 promotes the recruitment of immune cells and gut inflammation. Cell Rep..

[57] Chai L, Dai L, Che Y, Xu J, Liu G, Zhang Z, Yang R (2009). LRRC19, a novel member of the leucine-rich repeat protein family, activates NF-kappaB and induces expression of proinflammatory cytokines. Biochem. Biophys. Res. Commun..

[58] Wang YJ, Liu M, Jiang HY, Yu YW (2022). Downregulation of LRRC19 is associated with poor prognosis in colorectal cancer. J. Oncol..

[59] Zhang Y, Wang J, Liu X (2020). LRRC19-A bridge between selenium adjuvant therapy and renal clear cell carcinoma: A study based on datamining. Genes (Basel).

[60] Wang P, Sun B, Hao D, Zhang X, Shi T, Ma D (2010). Human TMEM174 that is highly expressed in kidney tissue activates AP-1 and promotes cell proliferation. Biochem. Biophys. Res. Commun..

[61] Sasaki S, Shiozaki Y, Hanazaki A, Koike M, Tanifuji K, Uga M, Kawahara K, Kaneko I, Kawamoto Y, Wiriyasermkul P, Hasegawa T, Amizuka N, Miyamoto KI, Nagamori S, Kanai Y, Segawa H (2022). Tmem174, a regulator of phosphate transporter prevents hyperphosphatemia. Sci. Rep..

[62] Zhang X, Hu F, Meng L, Gou L, Luo M (2014). Analysis of TMEM174 gene expression in various renal cancer types by RNA *in situ* hybridization. Oncol. Lett..

[63] Makhov P, Joshi S, Ghatalia P, Kutikov A, Uzzo RG, Kolenko VM (2018). Resistance to systemic therapies in clear cell renal cell carcinoma: Mechanisms and management strategies. Mol. Cancer Ther..

[64] Bejarano L, Jordāo MJC, Joyce JA (2021). Therapeutic targeting of the tumor microenvironment. Cancer Discov..

[65] Motzer RJ, Hutson TE, Tomczak P, Michaelson MD, Bukowski RM, Rixe O, Oudard S, Negrier S, Szczylik C, Kim ST, Chen I, Bycott PW, Baum CM, Figlin RA (2007). Sunitinib versus interferon alfa in metastatic renal-cell carcinoma. N. Engl. J. Med..

[66] Motzer RJ, Hutson TE, Cella D, Reeves J, Hawkins R, Guo J, Nathan P, Staehler M, de Souza P, Merchan JR, Boleti E, Fife K, Jin J, Jones R, Uemura H, De Giorgi U, Harmenberg U, Wang J, Sternberg CN, Deen K, McCann L, Hackshaw MD, Crescenzo R, Pandite LN, Choueiri TK (2013). Pazopanib versus sunitinib in metastatic renal-cell carcinoma. N. Engl. J. Med..

[67] Cornu M, Albert V, Hall MN (2013). mTOR in aging, metabolism, and cancer. Curr. Opin. Genet. Dev..

[68] Sun Y, Rha S, Lee SH, Guo J, Ueda T, Qin S, Naito S, Cincotta M, Tokushige K, Akaza H (2012). Phase II study of the safety and efficacy of temsirolimus in East Asian patients with advanced renal cell carcinoma. Jpn. J. Clin. Oncol..

[69] Han Y, Liu D, Li L (2020). PD-1/PD-L1 pathway: Current researches in cancer. Am. J. Cancer Res..

[70] Calvo E, Porta C, Grünwald V, Escudier B (2019). The current and evolving landscape of first-line treatments for advanced renal cell carcinoma. Oncologist.

[71] Shiravand Y, Khodadadi F, Kashani SMA, Hosseini-Fard SR, Hosseini S, Sadeghirad H, Ladwa R, O'Byrne K, Kulasinghe A (2022). Immune checkpoint inhibitors in cancer therapy. Curr. Oncol..

[72] Pan R, Pan F, Zeng Z, Lei S, Yang Y, Yang Y, Hu C, Chen H, Tian X (2022). A novel immune cell signature for predicting osteosarcoma prognosis and guiding therapy. Front. Immunol..

[73] Fu L, Bao J, Li J (2022). Crosstalk of necroptosis and pyroptosis defines tumor microenvironment characterization and predicts prognosis in clear cell renal carcinoma. Front. Immunol..

[74] Lin H, Fu L, Li P (2023). Fatty acids metabolism affects the therapeutic effect of anti-PD-1/PD-L1 in tumor immune microenvironment in clear cell renal cell carcinoma. J. Transl. Med..

[75] Bao JH, Li JB, Lin HS (2022). Deciphering a novel necroptosis-related miRNA signature for predicting the prognosis of clear cell renal carcinoma. Anal. Cell Pathol. (Amst.).

