# Supplementary figures and images for: Identification of a risk model for prognostic and therapeutic prediction in renal cell carcinoma based on infiltrating M0 cells
Source: Sci Rep. 2024 Jun 11;14:13390. doi: 10.1038/s41598-024-64207-0 (PMC11166996; doi:10.1038/s41598-024-64207-0)

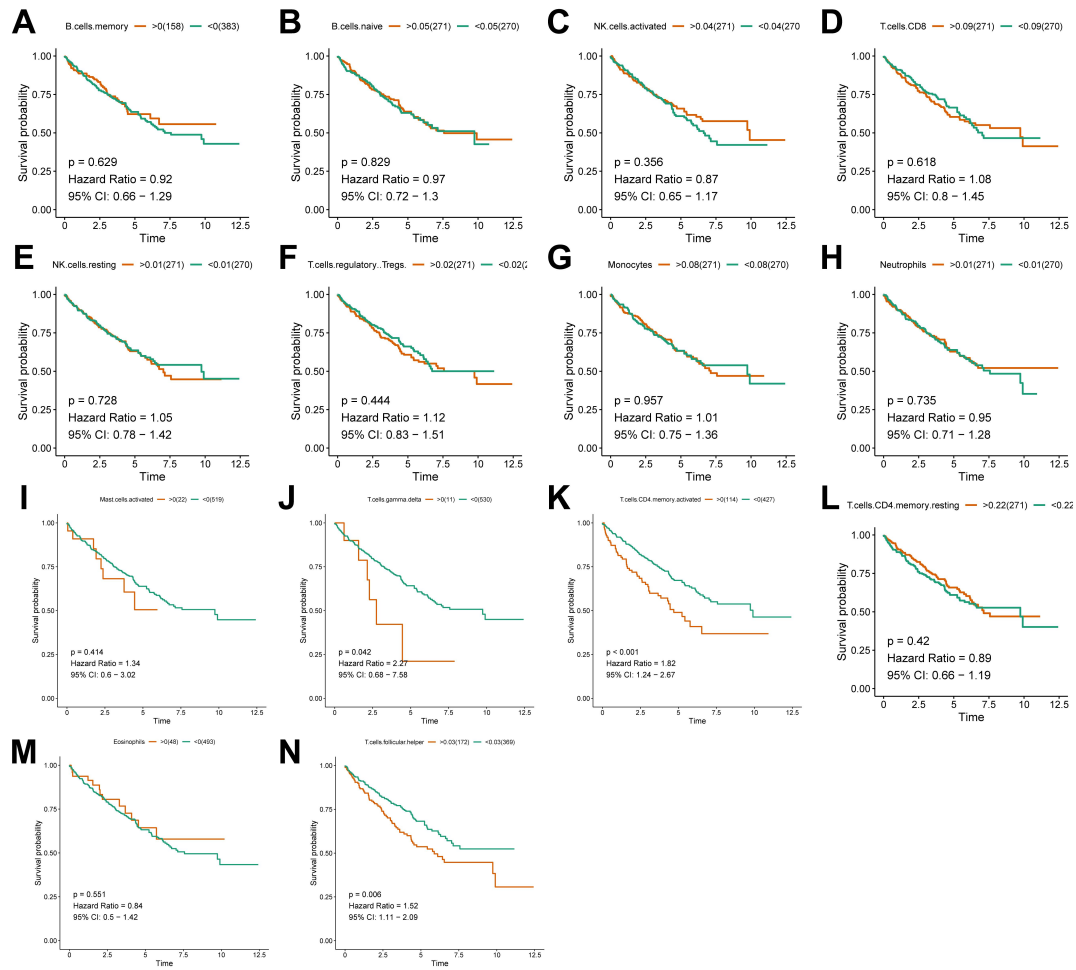

**Supplementary Figure 1. (A-N).**Results of KM survival analysis of fourteen types of immune cells.

Supplement: Supplementary file 7 — Supplementary Figure 1. [file 41598_2024_64207_MOESM7_ESM.pdf]

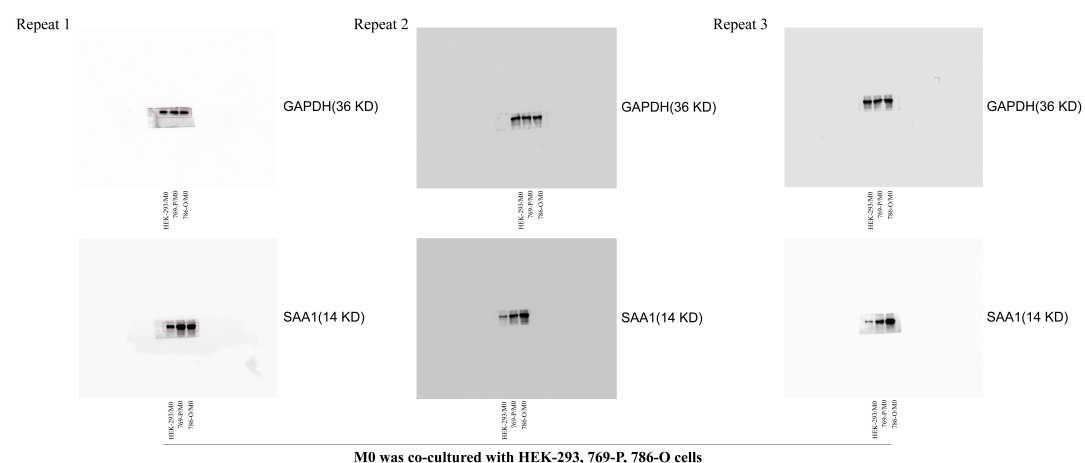

**Supplementary Figure 6.** The original Western blot figure. Repeat 1 results correspond to figure 12B.

Supplement: Supplementary file 12 — Supplementary Figure 6. [file 41598_2024_64207_MOESM12_ESM.pdf]

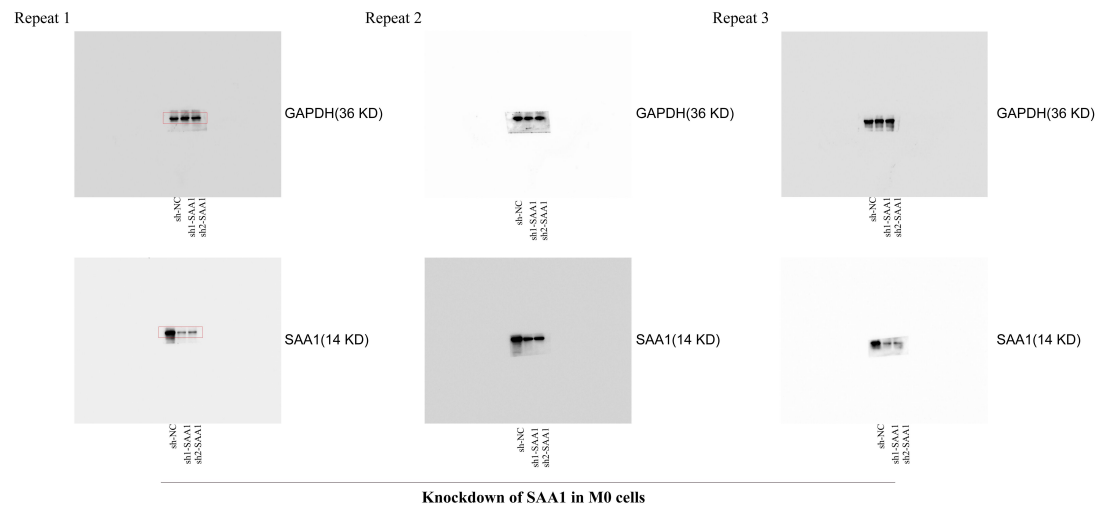

**Supplementary Figure 7.** The original Western blot figure. Repeat 1 results correspond to figure 12D.

Supplement: Supplementary file 13 — Supplementary Figure 7. [file 41598_2024_64207_MOESM13_ESM.pdf]
