# Supplementary material for: Identification of a risk model for prognostic and therapeutic prediction in renal cell carcinoma based on infiltrating M0 cells
Source: Sci Rep. 2024 Jun 11;14:13390. doi: 10.1038/s41598-024-64207-0 (PMC11166996; doi:10.1038/s41598-024-64207-0)
Supplement: Supplementary file 8 — Supplementary Figure 2. [file 41598_2024_64207_MOESM8_ESM.pdf]

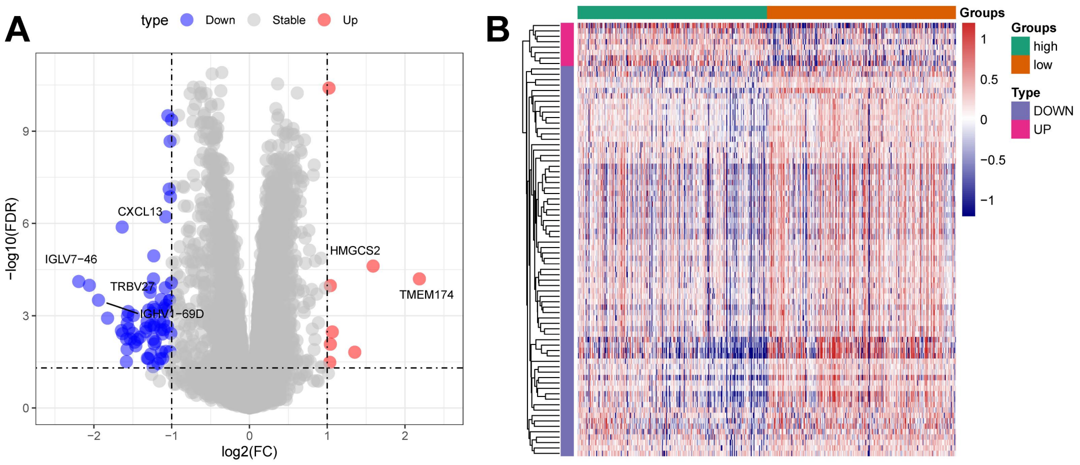

Supplementary Figure 2. (A). The volcano plot exhibited the DEGs between the high- and low-infiltrating groups of resting mast cells. **(B).**The heatmap exhibited DEGs between high- and low-infiltration groups of resting mast cells.
