# Supplementary material for: Identification of a risk model for prognostic and therapeutic prediction in renal cell carcinoma based on infiltrating M0 cells
Source: Sci Rep. 2024 Jun 11;14:13390. doi: 10.1038/s41598-024-64207-0 (PMC11166996; doi:10.1038/s41598-024-64207-0)
Supplement: Supplementary file 10 — Supplementary Figure 4. [file 41598_2024_64207_MOESM10_ESM.pdf]

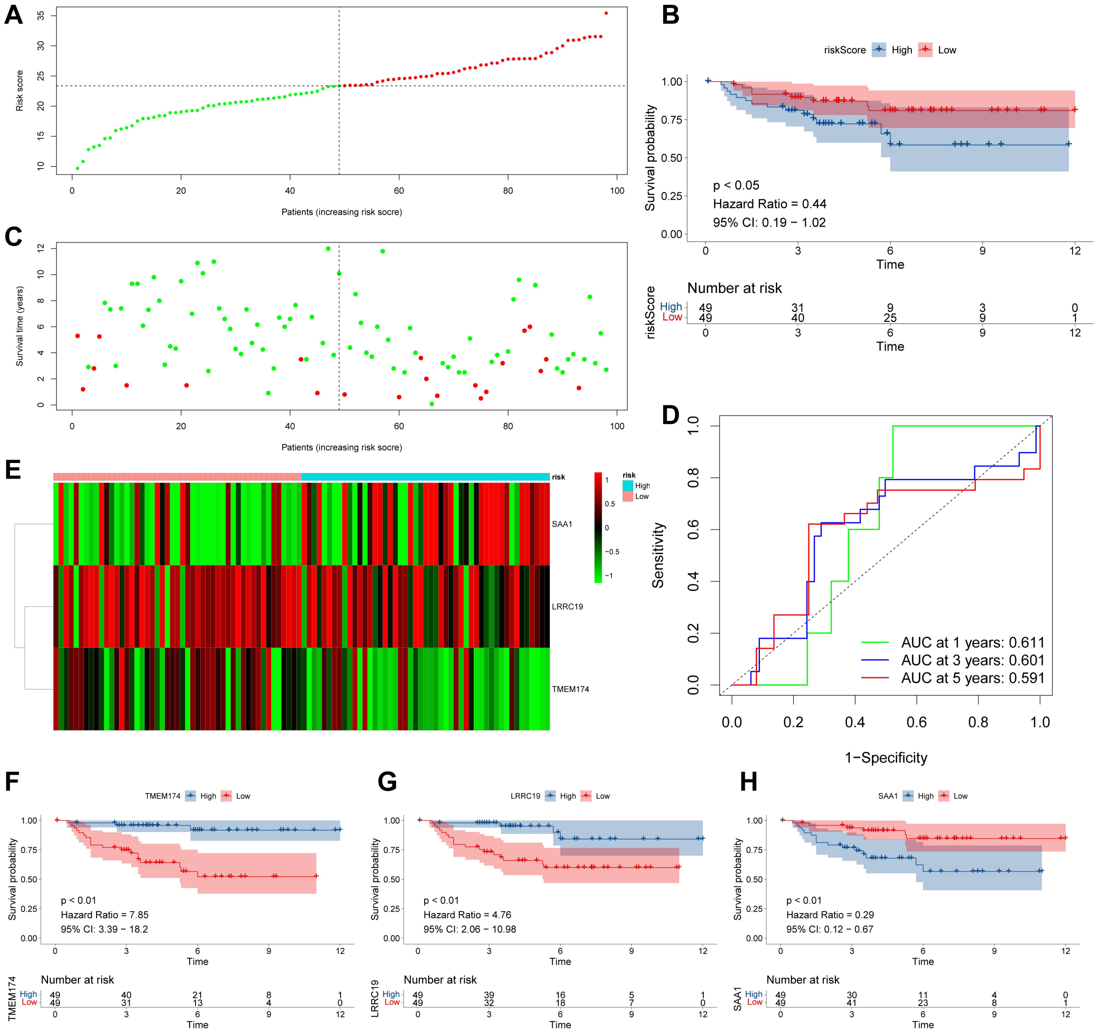

Supplementary Figure 4. (A). Samples in verification cohort were divided into low- and high-risk group according to median risk score. **(B).**KM survival analysis indicated the difference between LRG and HRG in verification cohort. **(C).**Alive and death cases between LRG and HRG in verification cohort. **(D).**ROC analysis of the risk model for the 1-year, 3-year and 5-year survival rates for ccRCC patients in verification cohort. **(E).**Expression of SAA1, LRRC19 and TMEM174 between LRG and HRG in verification cohort. **(F-H).**Survival analyses showed survival differences between the low and high expression groups of TMEM174, LRRC19 and SAA1 in the validation cohort.
