# Supplementary material for: Identification of a risk model for prognostic and therapeutic prediction in renal cell carcinoma based on infiltrating M0 cells
Source: Sci Rep. 2024 Jun 11;14:13390. doi: 10.1038/s41598-024-64207-0 (PMC11166996; doi:10.1038/s41598-024-64207-0)
Supplement: Supplementary file 11 — Supplementary Figure 5. [file 41598_2024_64207_MOESM11_ESM.pdf]

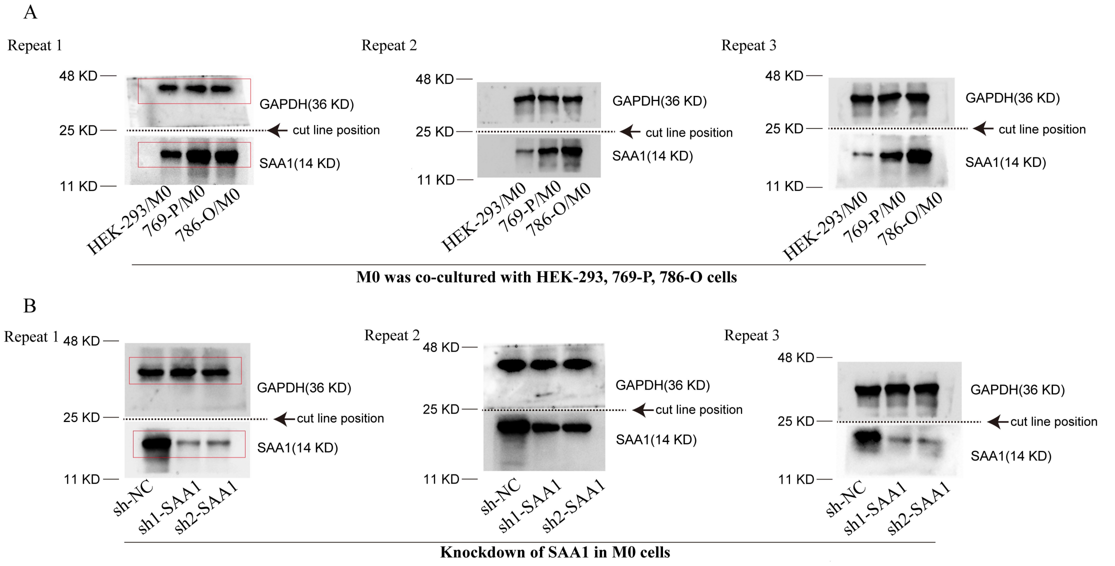

Supplementary Figure 5. (A).SAA1 expression in HEK-293 cells, 769-P cells, and 786-O cells after co-culture of each with M0 cells. **(B).**After knocking down SAA1 in M0 cells, they were co-cultured with tumor cells, and the effect on tumor cells was evaluated. Repeat 1, Repeat 2, and Repeat 3 represent triplicate results, respectively. The original images for all the repeated results shown in Supplementary Figure 5A are provided in Supplementary Figure 6, with a one-to-one correspondence in image ordering. The original images for all the repeated results shown in Supplementary Figure 5B are provided in Supplementary Figure 7, with a one-to-one correspondence in image ordering. The images marked by red boxes in parts A and B correspond to parts B and D in Figure 12.
