# Supplementary material for: Identification of a risk model for prognostic and therapeutic prediction in renal cell carcinoma based on infiltrating M0 cells
Source: Sci Rep. 2024 Jun 11;14:13390. doi: 10.1038/s41598-024-64207-0 (PMC11166996; doi:10.1038/s41598-024-64207-0)
Supplement: Supplementary file 9 — Supplementary Figure 3. [file 41598_2024_64207_MOESM9_ESM.pdf]

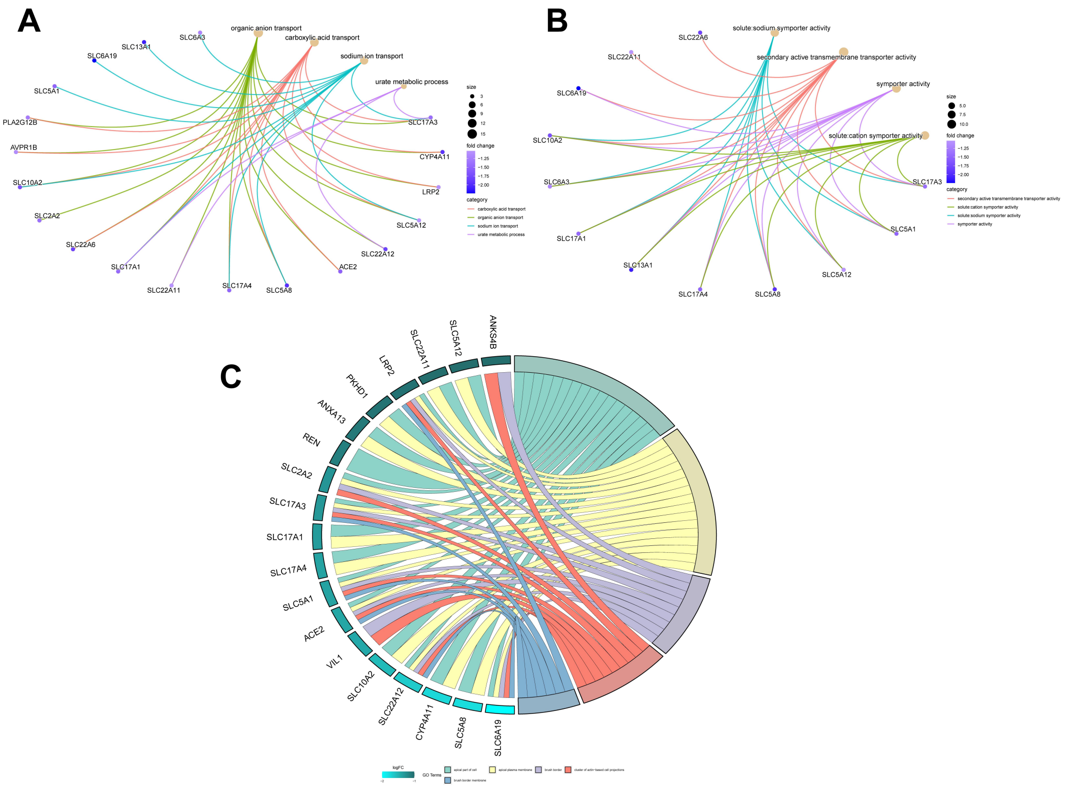

Supplementary Figure 3. (A-C).GO analyses (BP, CC, and MF) for the 39 genes associated with M0 macrophages.
